# Building the uracil skeleton in primitive ponds at the origins of life: carbamoylation of aspartic acid

**DOI:** 10.1038/s41598-022-21272-7

**Published:** 2022-11-10

**Authors:** Louis M. P. Ter-Ovanessian, Jean-François Lambert, Marie-Christine Maurel

**Affiliations:** 1 Laboratoire de Réactivité de Surface (LRS, UMR 7197 CNRS), Sorbonne Université, Case courrier 178, 4, Place Jussieu, 75005 Paris, France; 2 Institut de Systématique, Évolution, Biodiversité (ISYEB, UMR7205 CNRS), École Pratique Des Hautes Études, Muséum National d’Histoire Naturelle, Sorbonne Université, Université Des Antilles, CP 50, 57 rue Cuvier, 75005 Paris, France

**Keywords:** Astrobiology, Origin of life

## Abstract

A large set of nucleobases and amino acids is found in meteorites, implying that several chemical reservoirs are present in the solar system. The “geochemical continuity” hypothesis explores how protometabolic paths developed from so-called “bricks” in an enzyme-free prebiotic world and how they affected the origins of life. In the living cell, the second step of synthesizing uridine and cytidine RNA monomers is a carbamoyl transfer from a carbamoyl donor to aspartic acid. Here we compare two enzyme-free scenarios: aqueous and mineral surface scenarios in a thermal range up to 250 °C. Both processes could have happened in ponds under open atmosphere on the primeval Earth. Carbamoylation of aspartic acid with cyanate in aqueous solutions at 25 °C gives high N-carbamoyl aspartic acid yields within 16 h. It is important to stress that, while various molecules could be efficient carbamoylating agents according to thermodynamics, kinetics plays a determining role in selecting prebiotically possible pathways.

## Introduction

The question of the emergence of the first life forms, of which we know nothing and yet are the Darwinian descendants, can be tackled from the angle of the inert-to-living transition. The “geochemical continuity”^1^ hypothesis states that, at some stage during the evolution of life, key parts of metabolic pathways recapitulated reactions which previously occurred in a non-biological setting. It is both parsimonious and falsifiable, and also compatible with the idea that life developed in a continuous process rather than as a “freak accident”. Additionally, protometabolic paths from simple abundant precursors may continually resupply biochemical building blocks, avoiding the depletion problem encountered with exogenous delivery scenarios^2,3^. In this hypothesis, life-defining structures (metabolism, information, compartments) may have been initiated along the same general paths, but with other alternatives for kinetic (inorganic, including heterogeneous, catalysis) and thermodynamic control (free energy issued from macroscopic environment fluctuations) than those we observe today in organisms^4,5^.

In this line of thought, we are exploring a typical metabolic sequence of nucleotide biosynthesis, the de novo synthesis of pyrimidines (orotate pathway), with the aim to transpose it to an abiotic environment. Non-enzymatic pyrimidine biosynthesis has been the object of much interest lately, either through attempts at transposition of the orotate pathway^6^, or through alternative pathways involving different precursors^7^. In a previous publication, we considered the prebiotic potential of carbamoyl phosphate, an activated carbamoylating agent used at the beginning of this biochemical pathway. In the present one, we concentrate on the formation of N-carbamoyl-aspartic acid (NCA), the 7-atom precursor of the uracil framework.

NCA, also called ureidosuccinic acid^8^, exists in all living species, ranging from bacteria to eukaryotes. NCA is present in cytoplasm as well as excreta (saliva) and organs (prostate). It is synthesized from carbamoyl phosphate and L-aspartic acid through the action of the aspartate carbamoyltransferase enzyme (ATCase)^9^. As it plays a key role in aspartate and pyrimidine metabolisms, it is involved in several dysfunctions such as Canavan disease and dihydropyrimidinase deficiency^10^.

**Figure 1:**
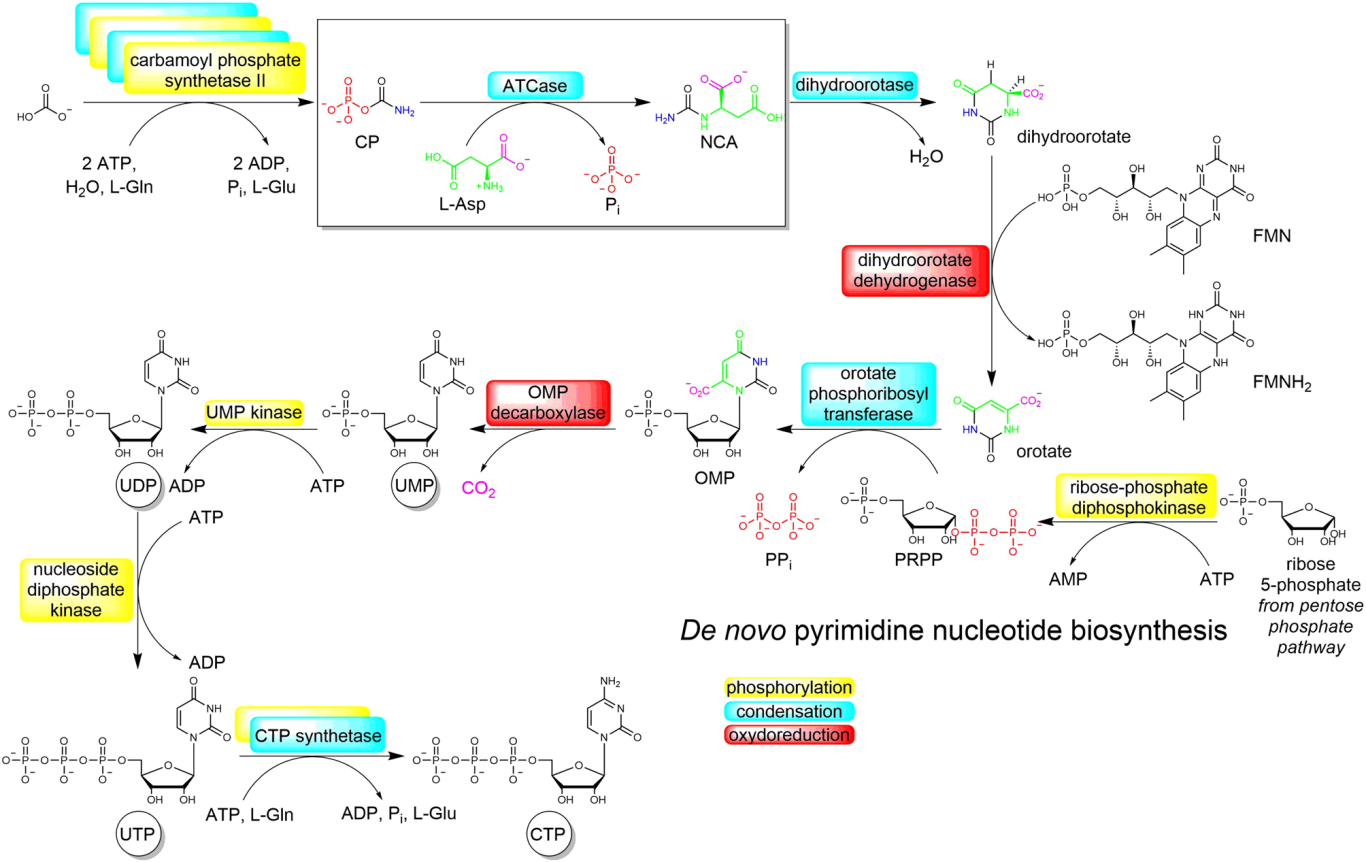
Current in vivo pyrimidine biosynthesis along the orotate pathway. The step studied here is boxed.

After an additional cyclization step, NCA forms the core skeleton of orotic acid^11,12^, the precursor of uracil^5,13,14^ (Fig. 1), hence it is a major prebiotic target to check whether the geochemical continuity hypothesis is valid.

**Figure 2:**
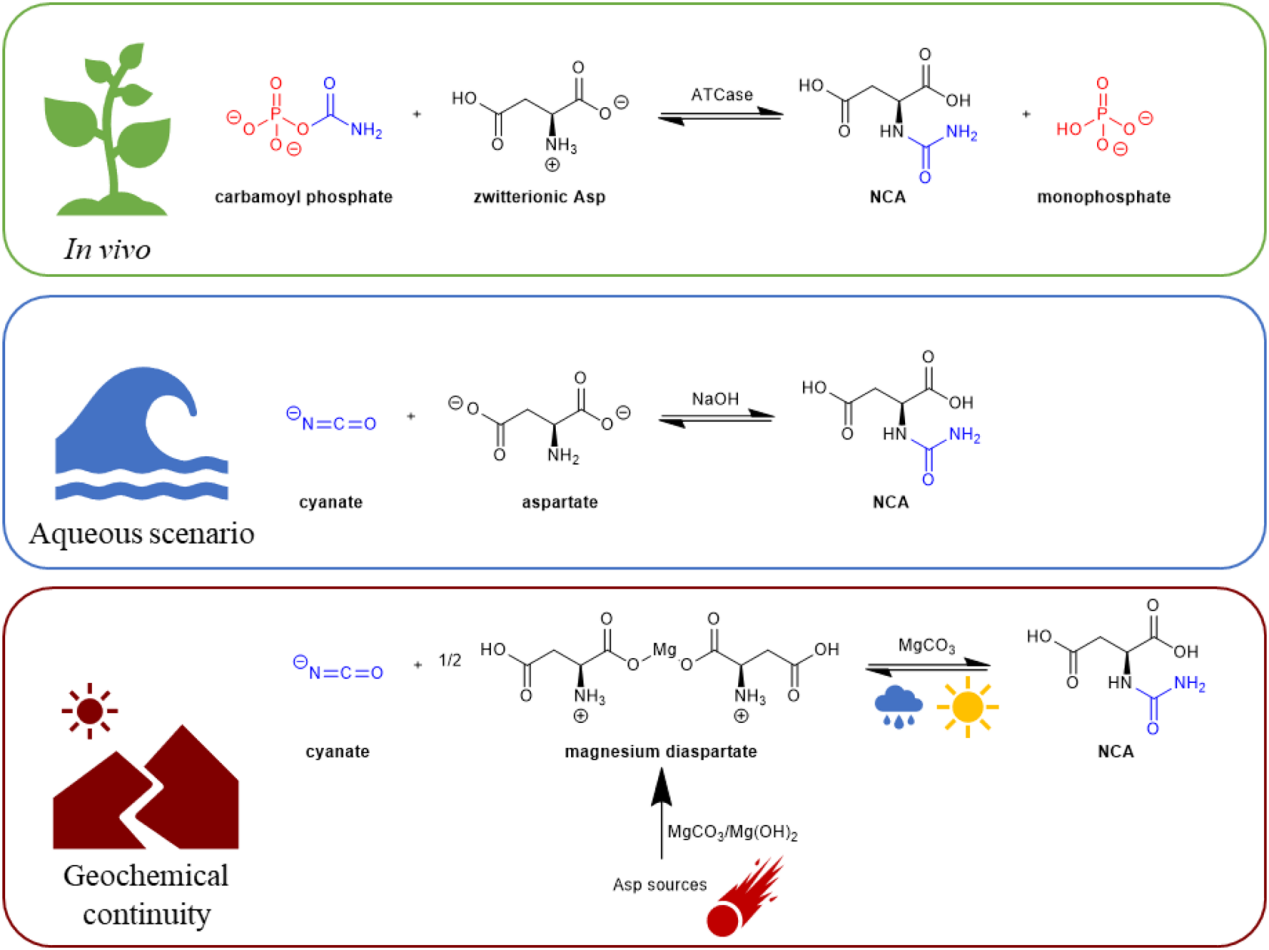
Three scenarios of NCA formation.

In previous work, we showed that carbamoyl phosphate (CP) is rather unstable under prebiotic conditions, but produces two other molecules still containing the energy-rich carbamoyl moiety: cyanate and urea^15^. It is therefore unlikely that CP itself was involved in a prebiotic carbamoylation pathway. However, the potential of cyanate and urea-type compounds as alternate carbamoylating agents deserves to be explored. Cyanate and urea-like compounds can be produced by several pathways in prebiotic settings, contrary to carbamoyl phosphate^16^. Therefore, we first investigated NCA synthesis through the reaction between cyanate and L-aspartic acid under alkaline aqueous conditions. Then, we also assessed mineral surface scenarios involving drying steps in order to test the predictions of the geochemical continuity hypothesis, including the idea that mineral catalysts can mimic the role of enzymes (Fig. 2).

## Results

### NCA formation in an aqueous scenario with cyanate

In our first experiment, the carbamoyl donor was sodium cyanate, reacting with aspartate under basic conditions (see Methods). The N-carbamoylation of aspartic acid has, in fact, been used long ago in Nyc’s protocol^17^ for orotic acid synthesis. The N-carbamoylation of generic amino acids by cyanate was further explored by the Commeyras team^18,19^.

**Figure 3:**
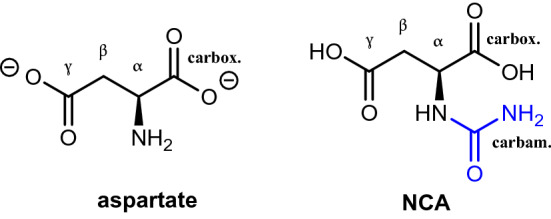
Comparison of the molecular structures of aspartate and of NCA. The added carbamoyl function is in blue.

The sodium hydroxide solution, initially containing aspartic acid and sodium cyanate in a 1:1 ratio, was left to react for 16 h at 25 °C, sampled and directly analysed in deuterated water by ^1^H NMR. Two sets of signals are identifiable (Fig. S1). The set of sodium aspartate (Fig. 3) is constituted by four signals corresponding to NH, H_α_ and the two H_β_ protons, the latter overlapped with signals of the second set. Similarly, the second set is comprised of four signals corresponding to NH, H_α_ and the two H_β_ protons, the latter overlapped with signals of the first set. This fits with NCA, a molecule that exhibits the same type of protons as aspartate, only slightly perturbed by carbamoylation.

The identification of the two sets is fully consistent with COSY correlation (Fig. S2). Minor peaks correlate as one set (Asp) whereas all major peaks (NCA) correlate together. In each set, H_α_ is correlated to H_β_ with a J^3^_H-H_. In addition, the two H_β_ are correlated to each other with a J^2^_H-H_. ^13^C NMR confirms the assignments (Fig. S3). A DEPT 135 acquisition (Fig. S4) discriminated the signals amongst each set. An extra ^13^C signal at 162.0 ppm with no correlation with hydrogen atoms was identified as a carbonate resulting from cyanate partial decomposition^15,20^. HSQC and HMBC correlations (Fig. S5 and S6) helped to link carbons and hydrogens without ambiguity. These results provide a clear spectroscopic signature of a successful NCA formation (Fig. 2).

By integrating proton signals, the average NCA yield after 16 h is 92%. Upon further evolution, it is found to be 88% after 23 days (Figs. S7 and S8) and 91% after one year (Fig. S9). Thus, the NCA remained stable (or rather metastable) for a long period of time, although the pH value drifted from 6.96 to 8.66, then 9.41, respectively. For comparison, using carbamoyl phosphate (instead of cyanate) and aspartate at 8.3 mM concentration and pH 8, Yi et al. obtained NCA in a 37% yield^6^.

### Absence of NCA formation in aqueous scenarios with urea and biuret

Two experiments were carried out under the same conditions (see Methods section) as the preceding one but cyanate was replaced by other potential carbamoyl donors.

**Figure 4:**
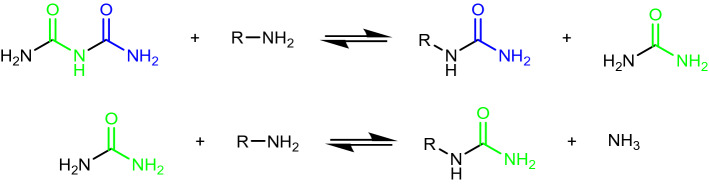
Biuret as a potential double carbamoyl donor.

Biuret, issued from the dimerization of urea^21–23^, is also relevant in prebiotic scenarios^24–27^. Theoretically, each biuret molecule could carbamoylate two aspartates following the two successive reactions shown in Fig. 4: thus, although the “carbamoylating agent” concentration is the same as in the preceding experiment, the carbamoyl group/aspartate ratio is 2:1 instead of 1:1.

After 16 h at 25 °C, the reaction products were analysed by ^1^H NMR (Fig. S10). Only one set of four signals was present and attributed to aspartate (Fig. 3). This lack of reactivity is confirmed by a COSY correlation (Fig. S11). It can be concluded that no detectable amount of NCA is formed in 16 h. Evolution was monitored for longer periods: after 23 days and even after 10 months, only unmodified aspartate was detected (Figs. S12 and S13).

Since the expected yield of this reaction at equilibrium would theoretically be 99.98%, based on the reaction Δ_r_G°’ (standard transformed molar free enthalpy of reaction, − 44.1 kJ/mol), we can conclude that the biuret-aspartate reaction has extremely slow kinetics. We cannot exclude that for very long time scale (longer than several years) carbamoylation by biuret could occur but, at any rate, kinetic competition would favour cyanate as a carbamoylating reagent—not to mention the fact that the rate of NCA decomposition by amide bond hydrolysis would be comparable to its rate of formation.

Urea was also checked as a potential carbamoylating agent^8^ but under the considered conditions (16 h, 25 °C), aspartate remained unmodified (Fig. S14).

### Asp dimerization in an aqueous scenario with activated phosphates

**Figure 5:**
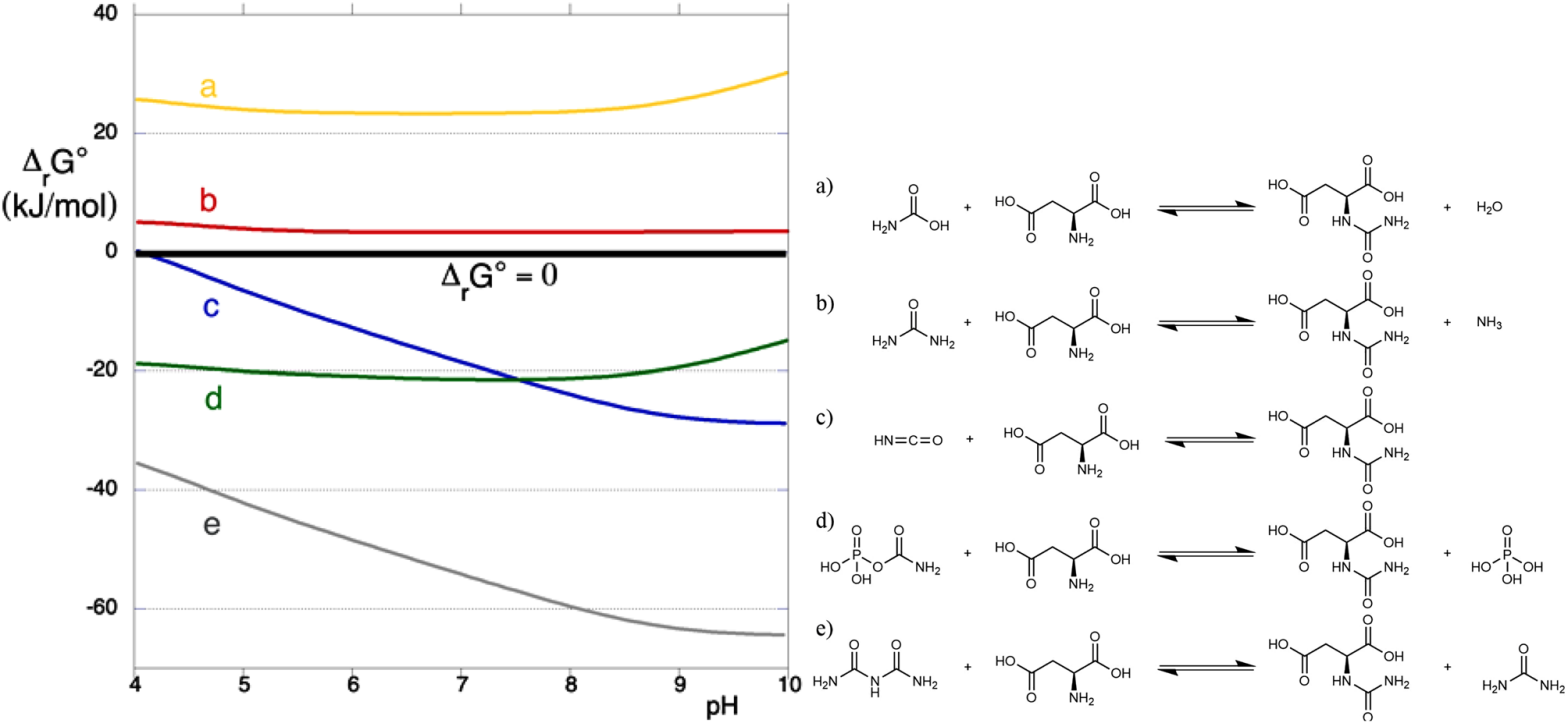
Left: pH dependence of transformed Gibbs free energy of reaction for biotic and abiotic NCA formation equilibria from aspartate and carbamate (**a**), urea (**b**), cyanate (**c**), carbamoyl phosphate (**d**), and biuret (**e**). The values are calculated for pMg = 5, ionic strength = 1 M. Right: the corresponding reactions, conventionally written with all reagents in the fully protonated form.

Carbamate itself (^-^O_2_C-NH_2_) cannot be an efficient carbamoylating agent because its free enthalpy content is too low: the calculated Δ_r_G°’ of NCA formation from carbamate and aspartate is largely positive at all pH (Fig. 5). One could have thought, however, that the addition of energy-rich reagents such as phosphorylating agents could allow aspartate carbamoylation by carbamate. Trimetaphosphate chemistry seems promising in this respect since this molecule and its degradation products have been widely used in prebiotic chemistry^28,29^; it has even been suggested that the “RNA world” was characterized by the use of trimetaphosphate as an energy source^30,31^.

To check this idea, a solution of phosphorylating agents (generated from trimetaphosphate ammonolysis at 70 °C for 66 h^15^) was reacted at 100 °C with L-aspartic acid and ammonium carbamate and analysed by ^1^H and ^13^C NMR. The targeted reaction—condensation of aspartic acid with carbamate—did not occur, but the initial signals of aspartate were nonetheless profoundly altered.

The doubling of each ^1^H NMR signal from L-aspartic acid alone can be interpreted as evidence of dimerization^32^. The split signals of L-aspartic acid present in ^1^H NMR could be unambiguously attributed to an Asp-Asp dimer (Figs. S15 and S16)^32^, that we assigned to the species β-Asp-Asp. Thermally obtained polyaspartic acid has a α/β ratio of 1/1.3^33^. Thermal polymerization is therefore hardly selective; in contrast, our reaction seems to form a single compound.

Thus, the free enthalpy contained in phosphoramidate was transferred to other high-energy bonds, namely the peptide bonds in the Asp-Asp dimer, affording another example of the versatility of phosphoramidates as coupling agents highlighted by Osumah and Krishnamurthy^34^; but it did not result in NCA formation.

### NCA on the rocks: preliminary studies on silica

Amorphous silica (SiO_2_) is well-known to be efficient for prebiotic condensation reactions, especially those of amino acids to peptides^35^, and is a prebiotically realistic mineral^36–38^. It is a moderately acidic catalyst, its catalytic properties relying on H-bonding and silanol acidity^39,40^. Thus, we co-adsorbed aspartate on silica with cyanate or urea, but also with carbamate and with ammonium carbonate. Most of these potential carbamoylating agents were eliminated by drying at room temperature, i.e., before they could react with the aspartate molecules, as revealed by transmission IR spectra of the dried systems (Fig. S17). For the (aspartate + cyanate), (aspartate + carbamate) and (aspartate + carbonate) systems, only the bands of adsorbed aspartate were observed, indicating that the other partner was lost to the gas phase. For the (aspartate + urea) on SiO_2_ system, the characteristic bands of urea are still visible after drying, but a subsequent TG + MS study indicated that urea decomposes thermally at 140 °C without reaction with aspartate.

### NCA on the rocks: thermal activations on magnesite

Since carbamoylation failed on acidic silica, we turned to a mineral support with basic properties. Brucite (Mg(OH)_2_) would be a candidate, already studied for aspartate adsorption and reactivity^41,42^. However, in the presence of a CO_2_-rich atmosphere, brucite is carbonated to magnesite (MgCO_3_)^43–47^, and so is periclase (MgO)^48^. Therefore, we chose magnesite as a support. Also, aspartic acid and Mg^2+^ ions from MgCO_3_ dissolution rapidly form the aspartate complex^49,50^, which prompted us to select Mg(Asp)_2_ for Asp deposition. Despite being marketed as MgCO_3_, powder XRD studies showed the mineral phase after impregnation and drying under nitrogen is always hydromagnesite (Mg_5_(CO_3_)_4_(OH)_2_·4H_2_O)^51,52^ (Fig. S19). No crystallisation of organic molecules was observed by XRD in biomolecules-impregnated hydromagnesite (Figs. S20, S22, S24 and S26).

After impregnation followed by drying at variable temperatures (Table S1), solid samples were directly desorbed with deuterated water. The desorption solutions were analysed by ^1^H (Figs. S25, S27, S28 and S29) and the spectra were compared with those of reference compounds (Figs. S18, S21 and S23). Hydromagnesite is partly soluble, so chances for organic molecules to stay adsorbed on the mineral and unanalyzed are negligible. According to ^1^H signal integration, a significant amount of NCA was already observable after drying at room temperature, and the NCA yield was improved by thermal treatment. An optimal yield seems to be reached at 150 °C (Fig. S27, Table S1). At 230 °C, new signals appeared, probably decomposition products of NCA and poly-Asp or poly-succinimide (Fig. S29).

Two reaction attempts of biuret and magnesium di-aspartate on magnesite were tried, at 25 °C and 140 °C. No condensation reaction occurred, as indicated by ^1^H NMR (Figs. S30 and S31).

## Discussion

### NCA formation in aqueous solution: thermodynamics and kinetics

The thermodynamic and kinetic parameters of biological compounds formation are considered with much care in many biochemical studies, especially in the two subdomains of bioenergetics and enzymatic catalysis. In contrast, they are often neglected in origins of life studies, although rigorous and insightful data have been published in some cases^53,54^.

A first rough indicator of whether a particular prebiotic reaction is thermodynamically possible is the standard (Gibbs) free energy of reaction, Δ_r_G°, or the transformed value at constant pH, Δ_r_G°’. Figure 5 displays the theoretically expected Δ_r_G°’ for NCA formation reactions from aspartate and several possible carbamoylating reagents in aqueous solution as a function of solution pH. These estimates permit differentiation between two sets of potential carbamoylating agents. With carbamate and urea, the reaction should be endergonic, although only slightly in the case of urea, and therefore these two molecules (especially carbamate) are not likely contenders for carbamoylation in solution. In contrast, aspartate carbamoylation by carbamoyl phosphate (the current biochemical agent), cyanate, and biuret should be exergonic. These values are pH-dependent, strongly so for biuret and cyanate; reaction with the latter becomes unfavourable at pH < 4, and conversely it is a more promising carbamoylating agent at high pH.

**Figure 6:**
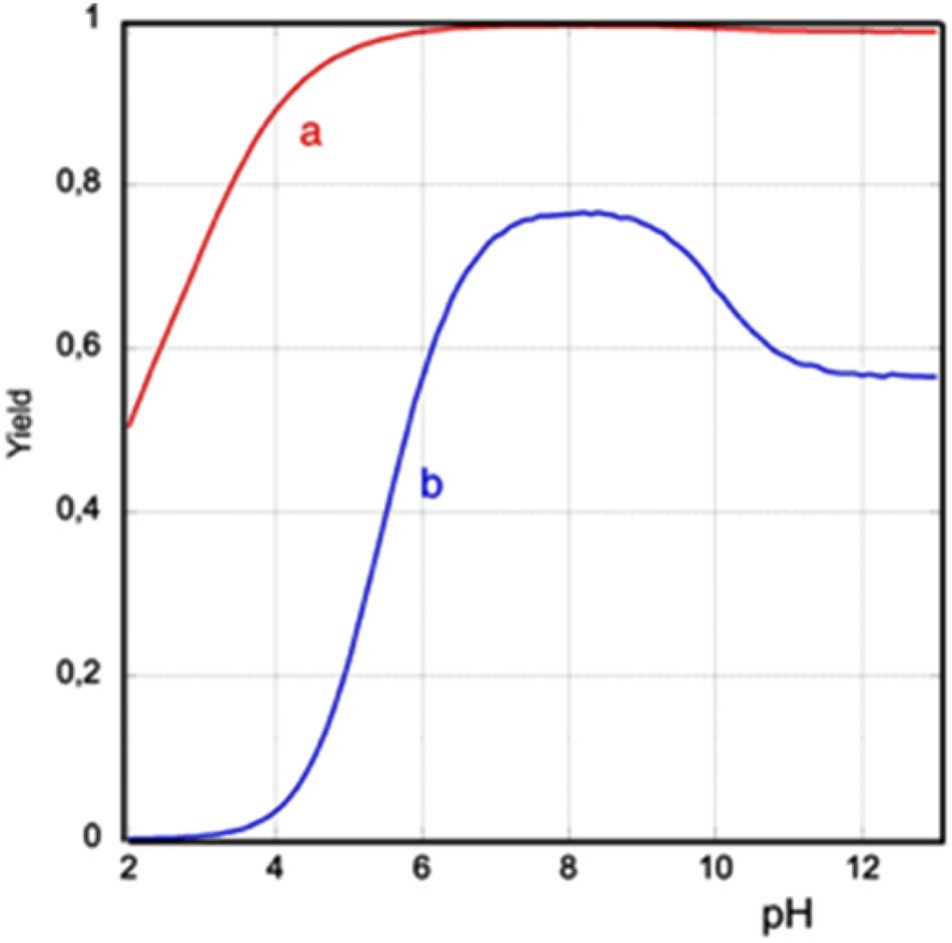
Theoretical equilibrium yield of NCA as a function of pH for (**a**) C°_Asp_ = C°_cyanate_ = 50 mmol.L^−1^, (**b**) C°_Asp_ = C°_cyanate_ = 25 µmol.L^−1^.

While the Δ_r_G° (resp. Δ_r_G°’) allow to calculate the equilibrium constants K (resp. K’), the equilibrium reaction yields will depend, in addition to the pH, on the initial concentrations of all reagents. For instance, reaction c) with cyanate is a simple condensation and should proceed further in the forward direction if the total concentrations are increased. As an illustration, Fig. 6 shows the expected NCA yields (at equilibrium) as a function of pH for an initial 1:1 molar ratio of cyanate to aspartate, and concentrations of 50 × 10^–3^ mol.L^-1^ (our conditions) and 25 × 10^–6^ mol.L^-1^.

In the conditions we used, the carbamoylation yield is expected to be at least 96% for all pH ≥ 5, and even in the micromolar range, it should be > 50% for pH ≥ 6. In the same way, excellent yields are expected using carbamoyl phosphate, and even more using biuret.

Experimentally, aspartate carbamoylation with cyanate results in a 92% yield after 16 h, and this composition remains stable for over a year. While this is not as high as the theoretically expected yields in the experimental pH range (> 99.3%), the reaction is indeed highly favoured, and seems to reach equilibrium in a few hours.

A very different situation is observed with biuret. While even higher carbamoylation yields would be expected, no reaction is detected even after 10 months. The kinetics of this reaction must therefore be extremely slow—no kinetic data were available in the literature to predict this conclusion. With urea, carbamoylation is expected to be only slightly endergonic, so that a > 30% yield would be predicted in our conditions. After 16 h, no reaction is observed, so this reaction is not very fast either.

In previous studies, Fox^55^ obtained NCA from Asp and urea in “hot water” with solid mineral bases. High yields (46–80%) were obtained; fast kinetics are the consequence of high temperatures, but certainly also of specific catalytic effects of the solid phases. Yamagata et al. reacted aspartic acid with urea at pH 7 and 90 °C, in an open flask that allowed for constant elimination of the NH_3_ product (open system)^56^. In these conditions, about 80% of the aspartate transformed into NCA, and further products formed from it, in a matter of days. This would mean that the carbamoylation reaction by urea, while slow at 25 °C, is reasonably fast at 90 °C.

In summary, aspartate carbamoylation is an illustration of the fact that in prebiotic chemistry, as in all branches of chemistry, kinetic and thermodynamic aspects must be considered in order to assess the feasibility of any given scenario. Thermodynamic data are available for many reactions; they tell us that carbamoylation by carbamate is not a feasible pathway, and by urea hardly so. Of course, an endergonic reaction can occur if it is coupled with an exergonic one, as happens often in biochemistry. However, such a coupling may only be considered if a convenient reaction pathway is available to induce coupling between the reagents: introducing the energy-rich trimetaphosphate in the system did allow an endergonic reaction, but it was aspartate dimerization instead of carbamoylation.

In contrast, thermodynamics tells us that both cyanate and biuret are possible carbamoylating agents. Here, kinetics comes into play. Usually kinetic studies have to be completely implemented since few such studies are available, even for biochemically important molecules. Only the cyanate reaction is promising from both the thermodynamic and kinetic points of view. We have not studied the biochemical carbamoylating agent, carbamoyl phosphate (CP), because a previous study has led us to conclude that it is unlikely to be present in prebiotic conditions, or, if formed, would probably isomerize to cyanate before reacting.

**Figure 7:**
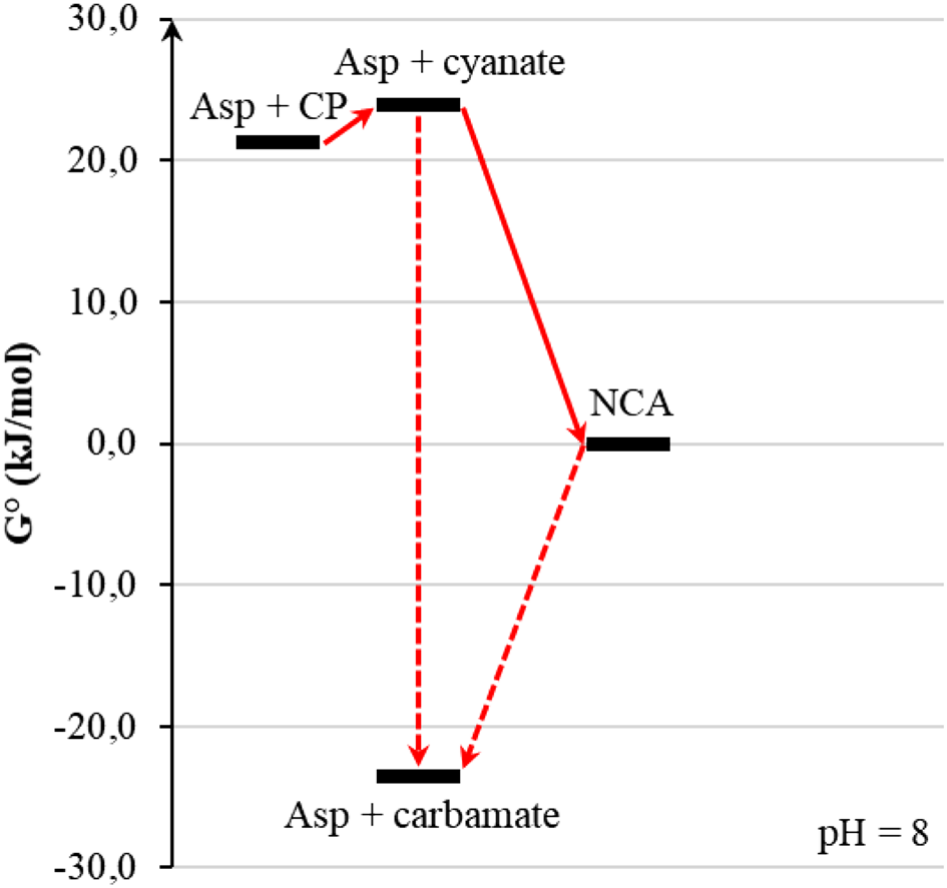
A partial energetic view of NCA formation reactions in aqueous solutions containing carbamoyl donors. Plain arrows correspond to fast transformations and dashed arrows to slower ones. Values based on *equilibrator.weizmann.ac.il* data.

Carbamoylation of aspartate by cyanate has all the characteristics of a “good” prebiotic reaction. It is strongly favoured because of a negative Δ_r_G°; there exists a fast reaction path even in the absence of specific catalysis, and its product, NCA, while still energy-rich, is kinetically inert (not prone to decomposition even on the scale of several months), so that it will remain available for futher transformations for a long time after being formed. This is illustrated in Fig. 7 where fast reactions are represented by full arrows and slow ones by dashed arrows.

This constitutes a simple example of how the elucidation of reaction thermodynamics and kinetics allows pruning a potentially complex network of prebiotic reaction pathways.

As mentioned, silica has been known to constitute a good platform for condensation reactions between molecules. The reason for this is twofold. First, from the thermodynamic point of view, silica allows to work in conditions of low water activity, by simply applying a drying step. Because water is a product of the condensation reaction, continuously removing it drags the condensation to the right according to Le Châtelier’s principle, and this is also true for any volatile condensation product. Therefore, carbamoylation by all the agents we considered could in principle be made favourable on silica (as opposed to the solution where only some agents are allowed). All it takes is for the surface to be thoroughly dehydrated, which in the case of fumed silica occurs at about 100 °C.

**Figure 8:**
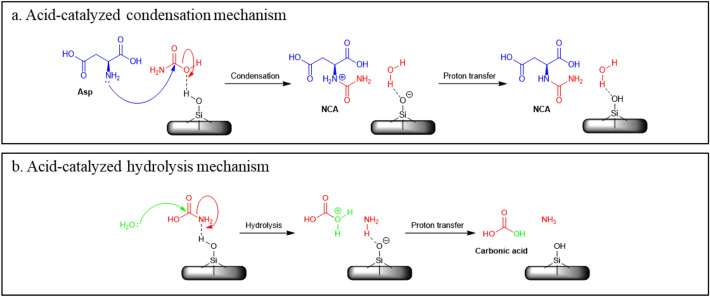
Silanol-assisted catalysis of carbamate condensation (**a**) and competitive hydrolysis (**b**) on silica.

Second, from the kinetic point of view, silica catalyses the nucleophilic addition that constitutes the first step of the condensation. Although in-depth studies have been devoted to the complexity of this phenomenon^57^, the basic mechanism probably implies weakly acidic surface silanols activating the leaving group (silica behaves as a weak solid Brönsted acid), as schematically illustrated in Fig. 8a for the aspartic acid/carbamic acid condensation.

It is a basic principle of kinetics that everything that catalyses a reaction in the forward direction also catalyses it in the reverse direction. For instance, if silica catalyses a condensation that liberates one water molecule, it will also catalyse the reverse reaction of hydrolysis: which one will actually occur depends on the water activity in the system. In fact, in the present case additional pathways may be considered for the reverse reaction as illustrated in Fig. 8b for the hydrolysis of carbamic acid.

Thus, we can understand the failure to observe carbamoylation reactions on silica. All carbamoylating agents already contain one C-N bond, which is usually quite labile. Silica is actually too good a catalyst: it will activate the C-N bond in the carbamoylating agent at low temperatures where water is still abundant on the surface, and thus cause its hydrolysis before it can functionalize the aspartic acid, a reaction that has a higher energy barrier.

Carbamoylating scenarios involving mineral surfaces are not completely excluded since NCA was observed from (cyanate + aspartate) deposited on MgCO_3_. We can say therefore that whatever catalytic sites are present on the MgCO_3_ surface (it would be expected to exhibit at least general basic catalysis), they are not able to significantly activate amide bonds at room temperature. They might still activate condensation at higher temperatures, as the NCA yields increase somewhat after heating at 150 °C, although remaining lower than those obtained in a mineral-free reaction. On the other hand, excessive heating causes a degradation of the NCA product, in a matter of hours at 230 °C: this represents the upper limit of the temperature range available for the use of the prebiotic precursor NCA.

## Conclusions

In this work, we have shown that among the various carbamoyl donors that could have been present in prebiotic conditions, cyanate is probably the most efficient to perform aspartate carbamoylation. This reaction has all the features of a “good” prebiotic reaction, and only necessitates precursors that are known to be formed prebiotically.

Our results underline the necessity of always considering both the thermodynamic and kinetic dimensions of potential prebiotic reactions. For instance, from a thermodynamic point of view, biuret should provide high carbamoylation yields, but the reaction rate at room temperature is so low that the corresponding scenario appears unlikely. In the same way, the input of chemical energy from a well-known inorganic precursor, trimetaphosphate, did result in energy transduction—but through the formation of an aspartate dimer, rather than the desired product, NCA. In this case, a different reaction pathway was opened.

While thermodynamic data on many biomolecules and their precursors are often available, at least in standard conditions, kinetic data are scarce and reaction kinetics must systematically be assessed.

The answer to the question asked at the beginning of this study—is aspartate carbamoylation a likely step in prebiotic pyrimidines synthesis?—is clearly positive, provided that cyanate is used as the carbamoylating agent. Thus, the geochemical continuity hypothesis is only partially vindicated^58^. While the reaction could very well have happened in prebiotic settings, it is likely to have used cyanate rather than the carbamoylating agent of current biochemistry, carbamoyl phosphate, which was probably not easily available^15^.

An aqueous phase scenario is quite likely. Mineral surfaces can actually be detrimental if they are acidic as is the case for silica. On the other hand, the basic hydromagnesite did not prevent carbamoylation by cyanate, but neither did it bring clear benefits. However, once NCA is formed, deposition on a mineral surface could stabilize it against thermal degradation, with respect to the species in solution. This aqueous solution/mineral surface versatility of the carbamoylation reaction could be part of a primitive pond scenario.

While we are trying to replicate the orotate pathway of extant biochemistry in a geochemical context, we do not want to rule out alternative sources of orotate. Indeed, Krishnamurthy et al. have investigated a different one-pot aqueous scenario^7^, in which orotate is obtained from hydantoin and glyoxylate. Several pathways to orotate may have once coexisted, with the current one being selected at some stage of chemical evolution.

In a forthcoming article, we will concentrate on replicating the next step in the orotate pathway, namely NCA cyclization, as it represents a key metabolic crossroads in current biochemistry.

## Methods

The following chemical compounds were purchased from commercial suppliers and used without further purification: ureidosuccinic acid (Sigma-Aldrich Co., cat. n°69037-500MG), biuret (Sigma-Aldrich Co., cat. n°15270-25G), L-aspartic hemimagnesium salt dihydrate (Sigma-Aldrich Co., cat. n°11260-100G), L-aspartic acid (Sigma-Aldrich Co., cat. n°A8949-100G), magnesium carbonate basic (Sigma-Aldrich Co., cat. n°13118-1 KG) with a Brunauer–Emmett–Teller surface area of 31.5 m^2^/g, sodium cyanate (Sigma-Aldrich Co., cat. n°185086-100G), sodium hydroxide anhydrous pellets (Carlo Erba reagents, cat. n°480507), deuterium oxide 99.90% D (Eurisotop, cat. n°D214FE), linear dimer H-Asp-Asp-OH (Bachem, cat. n°4010210.0250), ammonium carbamate (Sigma-Aldrich Co., cat. n°292834-100G), ammonia 28% analaR Normapur (VWR Chemicals, cat n°21190.292), trisodium trimetaphosphate (Sigma-Aldrich Co., cat. n°T5508-500G), urea ACS reagent (Sigma-Aldrich Co., cat. n°U5128-100G), ammonium carbonate ACS reagent (Aldrich chemical company, Inc., cat. n°20786-1), carbamoyl phosphate disodium salt (Sigma-Aldrich Co., cat. n°C4135-1G), fumed silica Aerosil 380 (Evonik Industries), with a Brunauer–Emmett–Teller surface area of 380 m^2^/g.

### Thermodynamics data

The Gibbs free energies of reactions were calculated by using the eQuilibrator calculator^59–63^ at http://equilibrator.weizmann.ac.il/.

### NMR experiments

NMR experiments were conducted on a Bruker Avance III 500 spectrometer (ω_L_ = 500.07 MHz for ^1^H and 125.74 MHz for ^13^C) equipped with a 5 mm inverse double resonance broadband probe. Chemical shifts were calibrated as δ values (ppm) relative to the peak of TMS set at δ = 0.00 ppm (^13^C NMR), residual light water in D_2_O set at δ = 4.79 ppm (^1^H NMR). Coupling constants are given in Hertz. All spectra were processed with the Bruker TopSpin 4.0.6 and 4.0.8 softwares. The used 2D correlations were the following: ^1^H-^1^H COSY (COrrelated SpectroscopY), ^1^H-^13^C HSQC (Heteronuclear Single Quantum Coherence) and ^1^H-^13^C HMBC (Heteronuclear Multiple Bond Correlation). The used DEPT 135 (Distortionless Enhancement by Polarization Transfer) experiment gives inverted CH_2_ and C groups.

pH measurements were carried out using a Fischer Scientific Accumet AE150 pH Benchtop Meter.

### XRD experiments

A small portion of each sample was ground and mounted on a zero-background holder. The X-ray powder diffraction data were registered at room temperature with a D8 DISCOVER Bruker diffractometer at Sorbonne University. This instrument is equipped with a Cu anode (Kα_1_ and Kα_2_ copper doublet) source operated at 40 kV and 30 mA and a LynxEye XE-T 1D detector. The data were recorded from 5° to 80° in 1 h with steps of 0.02°

### NCA Synthesis in aqueous solution

L-Aspartic acid (6.50 g, 50 mmol, 1 eq.) and sodium cyanate (3.21 g, 49 mmol, 1 eq.) were dissolved into a 1 M sodium hydroxide solution (50 mL). The resulting mixture was stirred and allowed to stand for 16 h at room temperature, then sampled (400 µL into 200 µL D_2_O) for NMR analyses.

### Asp + urea in aqueous solution

L-Aspartic acid (6.50 g, 50 mmol, 1 eq.) and urea (3.00 g, 50 mmol, 1 eq.) were dissolved into a 1 M sodium hydroxide solution (50 mL). The resulting mixture was stirred and allowed to stand for 16 h at room temperature, then sampled (400 µL into 200 µL D_2_O) for NMR analyses.

### Asp + biuret in aqueous solution

L-Aspartic acid (6.50 g, 50 mmol, 1 eq.) and biuret (5.08 g, 49 mmol, 1 eq.) were dissolved into a 1 M sodium hydroxide solution (50 mL). The resulting mixture was stirred and allowed to stand for 16 h at room temperature, then sampled (400 µL into 200 µL D_2_O) for NMR analyses.

### Asp + carbamate + phosphoramidates in ammonia solution

L-Aspartic acid (173 mg, 1.3 mmol, 1 eq.) and ammonium carbamate (132 mg, 1.7 mmol, 1.3 eq.) were dissolved into 2.5 mL of an ammonia solution containing phosphoramidates (sampled after 66 h at 70 °C, preparation described in^15^). The solution was stored in a 25 mL airtight bottle in an oven at 100 °C for 16 h. 600 µL were collected for NMR analyses.

### Deposition procedures

To deposit organic molecules on minerals, we used a wetting-and drying procedure, also called “incipient wetness impregnation” or IWI in the heterogeneous catalysis literature. It implies putting a solution containing an Asp precursor and carbamoyl donor in contact with a mineral surface to obtain a paste or slurry. During this process, solubilised organic compounds are in prolongated contact with the dispersed mineral and the slurry is subsequently dried at room temperature. Similar events could happen in a geochemical scenario due to climatic fluctuations.

### Asp + carbamoyl donors on SiO_2_

L-Aspartic acid (20 mg, 0.13 mmol, 1 eq.) and a carbamoyl donor* (0.20 mmol, 1.3 eq.) were dissolved into 5 mL distilled water under stirring. 500 mg of silica Aerosil were impregnated by the solution. The resulting slurry was dried under N_2_, until a dry powder was obtained. Additional treatment in a desiccator under vacuum removed water traces. The powder was ground and sampled for IR analyses. *urea (12 mg), ammonium carbonate (19 mg), ammonium carbamate (15 mg), sodium cyanate (13 mg) or sodium carbamoyl phosphate (27 mg).

### Mg(Asp)_2_ on MgCO_3_ reference

Magnesium di-aspartate (24 mg, 0.074 mmol, 2 eq. Asp) was dissolved into 6 mL distilled water under stirring. 500 mg (5.9 mmol, 39 eq.) of magnesium carbonate (5.9 mmol) were impregnated by the solution. The resulting suspension was dried under N_2_, until a dry powder was obtained. The powder was ground and sampled for XRD and NMR analyses (30 mg suspended into 600 µL D_2_O).

### Asp-Asp on MgCO_3_ reference

Asp-Asp dimer (24 mg, 0.15 mmol) was dissolved into 6 mL distilled water under stirring. 500 mg of magnesium carbonate were impregnated by the solution. The resulting suspension was dried under N_2_, until a dry powder was obtained. The powder was ground and sampled for XRD and NMR analyses (30 mg suspended into 600 µL D_2_O).

### NCA on MgCO_3_ reference

Ureidosuccinic acid (22 mg, 0.12 mmol) was dissolved into 6 mL distilled water under stirring. 500 mg of magnesium carbonate (5.9 mmol) were impregnated by the solution. The resulting suspension was dried under N_2_, until a dry powder was obtained. The powder was ground and sampled for XRD and NMR analyses (30 mg suspended into 600 µL D_2_O).

### Mg(Asp)_2_ + cyanate on MgCO_3_

Magnesium di-aspartate (24 mg, 0.074 mmol, 2 eq. Asp) and sodium cyanate (13 mg, 0.20 mmol, 1.4 eq.) were dissolved into 6 mL distilled water under stirring. 500 mg (5.9 mmol, 39 eq.) of magnesium carbonate (5.9 mmol) were impregnated by the solution. The resulting suspension was dried under N_2_, until a dry powder was obtained. The powder was ground and sampled for thermal activations. 10 mg of the powder were thermally activated at four different temperatures (150 °C, 200 °C and 230 °C) for 30 min in an oven fitted with desiccants. Hygrometry measurement at 88 °C is 3.5% RH. After cooling down in a desiccator under reduced pressure, the samples were analysed by NMR (10 mg suspended into 600 µL D_2_O).

### Mg(Asp)_2_ + biuret on MgCO_3_

Magnesium di-aspartate (24 mg, 0.074 mmol, 2 eq. Asp) and biuret (21 mg, 0.20 mmol, 1.4 eq.) were dissolved into 6 mL distilled water under stirring. 500 mg (5.9 mmol, 39 eq.) of magnesium carbonate (5.9 mmol) were impregnated by the solution. The resulting suspension was dried under N_2_, until a dry powder was obtained. The powder was grinded and sampled for thermal activations. 10 mg of the powder were thermally activated at 140 °C for 30 min into an oven packed with desiccants. Hygrometry measurement at 88 °C is 3.5% RH. After cooling down into a reduced pressure desiccator, samples were analysed by NMR (10 mg suspended into 600 µL D_2_O).

## Supplementary Information

Supplementary Information.

## Data Availability

The authors declare that all data supporting the findings of this study are available within the paper and Supplementary Information.
