# Supplementary material for: Building the uracil skeleton in primitive ponds at the origins of life: carbamoylation of aspartic acid
Source: Sci Rep. 2022 Nov 10;12:19178. doi: 10.1038/s41598-022-21272-7 (PMC9649776; doi:10.1038/s41598-022-21272-7)
Supplement: Supplementary file 1 — Supplementary Information. [file 41598_2022_21272_MOESM1_ESM.docx]

Supplementary material for article

Building the uracil skeleton in primitive ponds at the origins of life: carbamoylation of aspartic acid

**Louis M.P. Ter-Ovanessian^1^, Jean-François Lambert^2^ (corresponding author) and Marie-Christine Maurel^3^**

*^1^ LRS & ISYEB, Sorbonne Université, CNRS, 75005 Paris, France. louis.ter_ovanessian@sorbonne-universite.fr*

*^2^ Laboratoire de Réactivité de Surface (LRS), Sorbonne Université, CNRS, F-75005 Paris, France. jean-francois.lambert@sorbonne-universite.fr*

*^3^* *Institut de Systématique, Évolution, Biodiversité (ISYEB), Muséum national d'Histoire naturelle, Sorbonne Université, École Pratique des Hautes Études, Université des Antilles, CNRS ; CP 50, 57 rue Cuvier 75005 Paris, France. marie-christine.maurel@sorbonne-universite.fr.*

Table of contents

| **Abbreviations and chemical nomenclature** | **SI 01** |
| --- | --- |

**Results**

| 1. Asp + sodium cyanate + sodium hydroxide solution after 16 h at 25 °C | SI 02 |
| --- | --- |
| 1. Asp + sodium cyanate + sodium hydroxide solution after 23 days at 25 °C | **SI 08** |
| 1. Asp + sodium cyanate + sodium hydroxide solution after 1 year at 25 °C | **SI 10** |
| 1. Asp + urea + sodium hydroxide solution after 16 h at 25 °C | **SI 11** |
| 1. Asp + biuret + sodium hydroxide aqueous solution after 16 h at 25 °C | **SI 12** |
| 1. Asp + biuret + sodium hydroxide aqueous solution after 23 days at 25 °C | **SI 14** |
| 1. Asp + biuret + sodium hydroxide aqueous solution after 10 months at 25 °C | **SI 15** |
| 1. Asp + carbamate + phosphoramidate aqueous solution after 16 h at 100 °C | **SI 16** |
| 1. Asp + carbamoyl donors on silica at 25 °C | **SI 18** |
| 1. Mg(Asp)_2_ on magnesium carbonate at 25 °C | **SI 19** |
| 1. Asp-Asp on magnesium carbonate at 25 °C | **SI 21** |
| 1. NCA reference spectra | **SI 23** |
| 1. Mg(Asp)_2_ + sodium cyanate on magnesium carbonate at 25 °C | **SI 25** |
| 1. Mg(Asp)_2_ + sodium cyanate on magnesium carbonate at 150 °C | **SI 27** |
| 1. Mg(Asp)_2_ + sodium cyanate on magnesium carbonate at 200 °C | **SI 28** |
| 1. Mg(Asp)_2_ + sodium cyanate on magnesium carbonate at 230 °C | **SI 29** |
| 1. Mg(Asp)_2_ + biuret on magnesium carbonate at 25 °C | **SI 30** |
| 1. Mg(Asp)_2_ + biuret on magnesium carbonate at 140 °C | **SI 31** |

**Abbreviations and chemical nomenclature**

Asp: L-aspartic acid or aspartate

Asp_2_, Asp-Asp or H-Asp-Asp-OH: linear dimer of L-aspartic acid

Mg(Asp)_2_: Aspartate complex

NCA: N-carbamoyl-aspartic acid

**Results**

**1) Asp + sodium cyanate + sodium hydroxide aqueous solution after 16 h at 25 °C**

pH measurements:

Initial: 6.96. After 16 h: 8.66. After 377 days: 9.41.

NMR data:

^1^H NMR (Bruker, 500.07 MHz, D_2_O, 25 °C, ppm)

Aspartate: δ 6.29 (s, NH); 3.73 (ddd, 1.3 Hz, 4.1 Hz, 8.4 Hz, 1H, H_α_); 2.66 (dd, 4.0 Hz, 17.2 Hz, 1H, H_β1_); 2.52 (dd overlapped with NCA, 4.0 Hz, 17.2 Hz, 1H, H_β2_)

N-carbamoylaspartate: δ 5.53 (s, NH); 4.12 (m, 1H, H_α_), 2.54 (dd overlapped, 4.0 Hz, 15.4 Hz, H_β1_); 2.34 (dd, 9.6 Hz, 15.4 Hz, 1H, H_β2_)


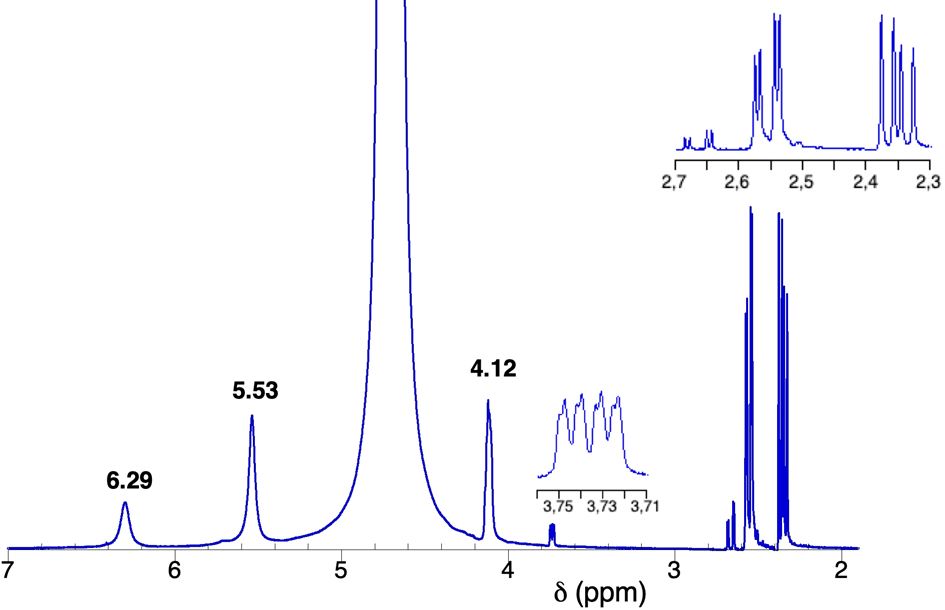


**Fig. S1** ^1^H NMR spectrum of Asp + sodium cyanate + sodium hydroxide aqueous solution after 16 h at 25 °C

COSY (Bruker, 500.07 and 500.07 MHz, D_2_O, 25 °C, ppm)


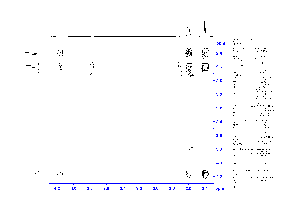


**Fig. S2** COSY 2D NMR spectrum of Asp + sodium cyanate + sodium hydroxide aqueous solution after 16 h at 25 °C

^13^C NMR (Bruker, 125.7 MHz, D_2_O, 25 °C, ppm)

Aspartate: δ 178.2 (s, C_carbox_); 175.9 (s, C_γ_) 52.6 (s, C_α_); 37.9 (s, C_β_)

N-carbamoylaspartate: δ 179.9 (s, C_carbox_); 179.3 (s, C_γ_); 161.0 (s, C_carbam_); 53.8 (s, C_α_); 40.4 (s, C_β_)

Carbonate: δ 162.0 (s)


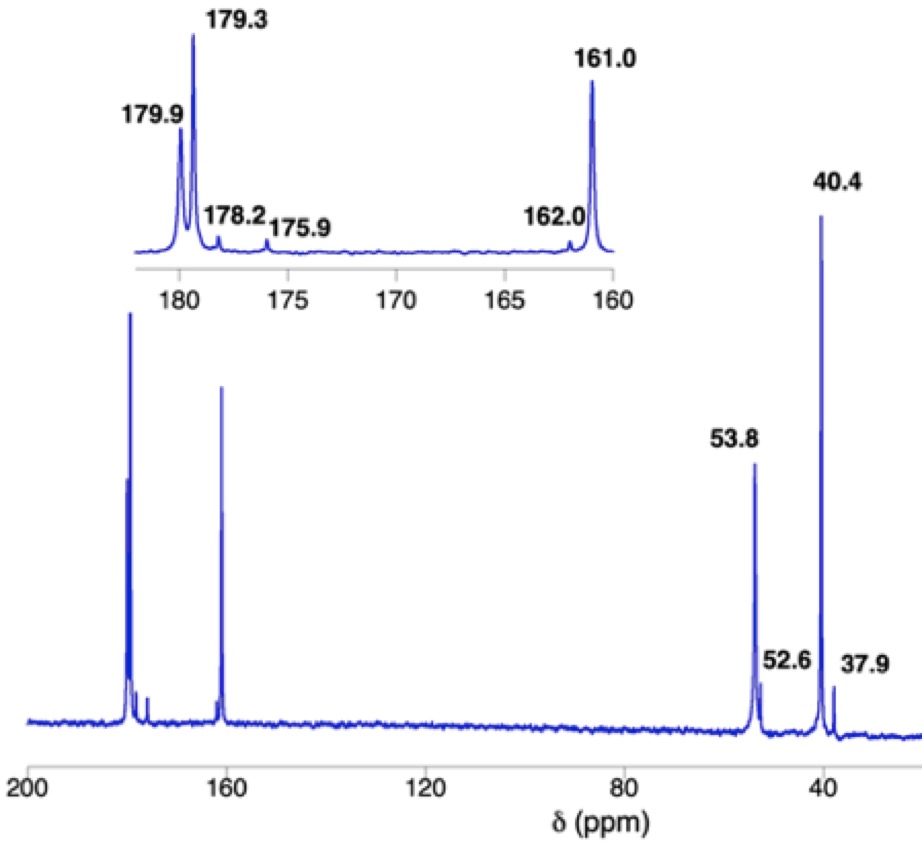


**Fig. S3** ^13^C NMR spectrum of Asp + sodium cyanate + sodium hydroxide aqueous solution after 16 h at 25 °C

DEPT 135 (Bruker, 125.7 MHz, D_2_O, 25 °C, ppm)

Aspartate: δ 52.6 (CH, C_α_); 37.9 (CH_2_, C_β_)

N-carbamoylaspartate: δ 53.7 (CH, C_α_); 40.4 (CH_2_, C_β_)

Quaternary carbons are not visible.


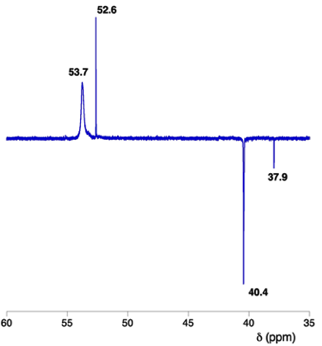


**Fig. S4** ^13^C DEPT 135 NMR spectrum of Asp + sodium cyanate + sodium hydroxide aqueous solution after 16 h at 25 °C

HSQC (Bruker, 500.07 and 125.7 MHz, D_2_O, 25 °C, ppm)

**Fig. S5** HSQC 2D NMR spectrum of Asp + sodium cyanate + sodium hydroxide aqueous solution after 16 h at 25 °C

HMBC (Bruker, 500.07 and 125.7 MHz, D_2_O, 25 °C, ppm)

**Fig. S6** HMBC 2D NMR spectrum of Asp + sodium cyanate + sodium hydroxide aqueous solution after 16 h at 25 °C

**2) Asp + sodium cyanate + sodium hydroxide aqueous solution after 23 days at 25 °C**

NMR data:

^1^H NMR (Bruker, 500.07 MHz, D_2_O, 25 °C, ppm)

Aspartate: δ 6.32 (s, NH); 3.78 (dd, 4.0 Hz, 8.2 Hz, 1H, H_α_); 2.70 (dd, 4.0 Hz, 17.1 Hz, 1H, H_β1_); 2.57 (dd overlapped with NCA, 1H, H_β2_)

N-carbamoylaspartate: δ 5.57 (s, NH); 4.17 (m, 1H, H_α_), 2.59 (dd overlapped, 4.0 Hz, 15.4 Hz, H_β1_); 2.39 (dd, 9.6 Hz, 15.4 Hz, 1H, H_β2_)

**
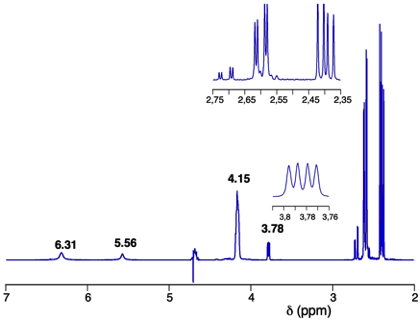
**

**Fig. S7** ^1^H NMR spectrum of Asp + sodium cyanate + sodium hydroxide aqueous solution after 23 days at 25 °C

COSY (Bruker, 500.07 and 500.07 MHz, D_2_O, 25 °C, ppm)

**
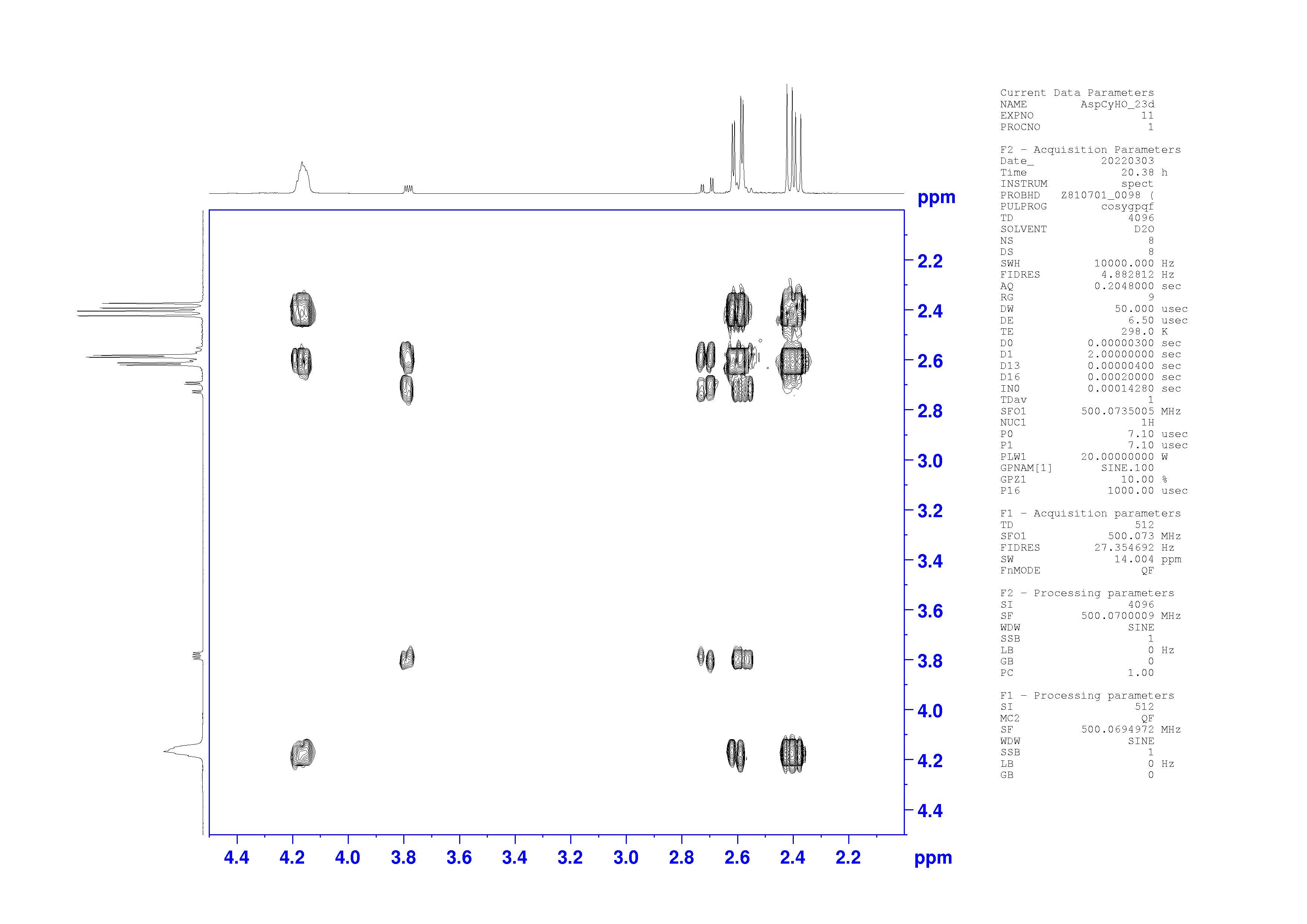
**

**Fig. S8** COSY 2D NMR spectrum of Asp + sodium cyanate + sodium hydroxide aqueous solution after 23 days at 25 °C

**3) Asp + sodium cyanate + sodium hydroxide aqueous solution after 1 year at 25 °C**

pH measurements:

After 379 days: 9.41.

NMR data:

^1^H NMR (Bruker, 500.07 MHz, D_2_O, 25 °C, ppm)

Aspartate: δ 6.32 (s, NH); 3.78 (dd, 4.0 Hz, 8.2 Hz, 1H, H_α_); 2.70 (dd, 3.8 Hz, 16.9 Hz, 1H, H_β1_); 2.57 (dd overlapped with NCA, 1H, H_β2_)

N-carbamoylaspartate: δ 5.57 (s, NH); 4.17 (m, 1H, H_α_), 2.59 (dd overlapped, 4.0 Hz, 15.4 Hz, H_β1_); 2.39 (dd, 9.7 Hz, 15.4 Hz, 1H, H_β2_)

**
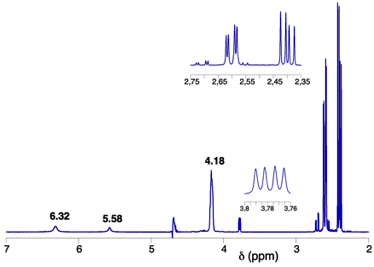
**

**Fig. S9** ^1^H NMR spectrum of Asp + sodium cyanate + sodium hydroxide aqueous solution after 1 year at 25 °C

**4) Asp + urea + sodium hydroxide solution after 16 h at 25 °C**

NMR data:

^1^H NMR with solvent suppression (Bruker, 500.07 MHz, D_2_O, 25 °C, ppm)

Aspartate: δ 3.84 (dd, 4.0 Hz, 8.4 Hz, 1H, H_α_); 2.75 (dd, 3.9 Hz, 17.5 Hz, 1H, H_β1_); 2.64 (dd, 8.4 Hz, 17.5 Hz, H_β2_)

**
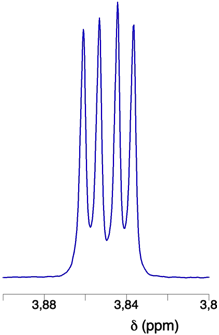

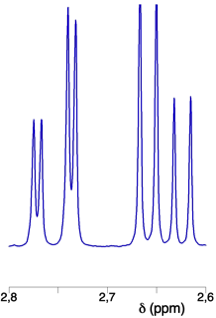
**

**Fig. S10** ^1^H NMR spectrum of Asp + urea + sodium hydroxide aqueous solution after 16 h at 25 °C

**5)** **Asp + biuret + sodium hydroxide aqueous solution after 16 h at 25 °C**

pH measurements:

Initial: 5.22. After 16 h: 5.30. After 279 days: 5.57.

NMR data:

^1^H NMR with solvent suppression (Bruker, 500.07 MHz, D_2_O, 25 °C, ppm)

Aspartate: δ 6.76 (s, NH, possibly from biuret); 3.80 (dd, 3.9 Hz, 8.3 Hz, 1H, H_α_); 2.71 (dd, 3.9 Hz, 17.6 Hz, 1H, H_β1_); 2.59 (dd, 8.3 Hz, 17.5 Hz, H_β2_)


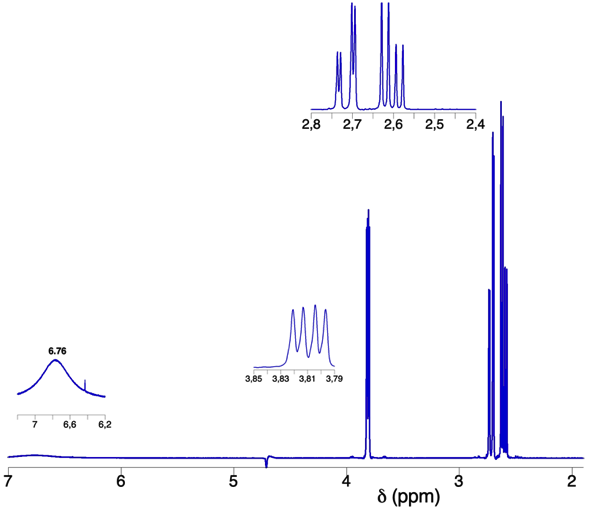


**Fig. S11** ^1^H NMR spectrum of Asp + biuret + sodium hydroxide aqueous solution after 16 h at 25 °C

COSY (Bruker, 500.07 and 500.07 MHz, D_2_O, 25 °C, ppm)

**Fig. S12** COSY 2D NMR spectrum of Asp + biuret + sodium hydroxide aqueous solution after 16 h at 25 °C

**6) Asp + biuret + sodium hydroxide aqueous solution after 23 days at 25 °C**

NMR data:

^1^H NMR with solvent suppression (Bruker, 500.07 MHz, D_2_O, 25 °C, ppm)

Aspartate: δ 6.79 (s, NH, possibly from biuret); 3.85 (m (ddd?), 2.2 Hz, 8.3 Hz, 1H, H_α_); 2.75 (ddd, 2.1 Hz, 3.8 Hz, 17.6 Hz, 1H, H_β1_); 2.64 (ddd, 2.2 Hz, 8.5 Hz, 17.6 Hz, H_β2_)

**
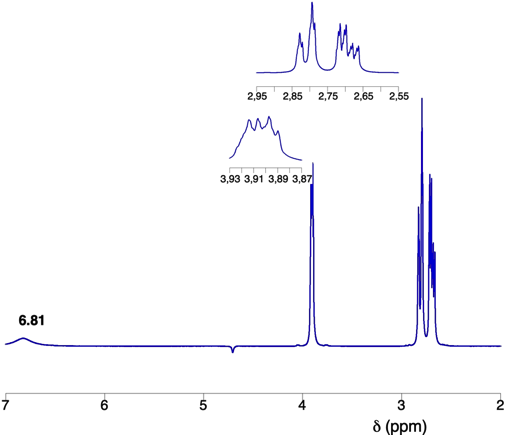
**

**Fig. S13** ^1^H NMR spectrum of Asp + biuret + sodium hydroxide aqueous solution after 23 days at 25 °C

**7) Asp + biuret + sodium hydroxide aqueous solution after 10 months at 25 °C**

pH measurements:

After 277 days: 5.57

NMR data:

^1^H NMR with solvent suppression (Bruker, 500.07 MHz, D_2_O, 25 °C, ppm)

Aspartate: δ 6.79 (s, NH, possibly from biuret); 3.85 (dd, 3.9 Hz, 8.5 Hz, 1H, H_α_); 2.75 (dd, 3.8 Hz, 17.4 Hz, 1H, H_β1_); 2.63 (dd, 8.4 Hz, 17.5 Hz, H_β2_)

**
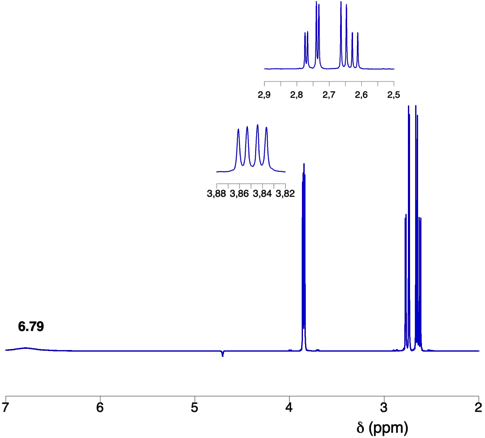
**

**Fig. S14** ^1^H NMR spectrum of Asp + biuret + sodium hydroxide aqueous solution after 10 months at 25 °C

**8) Asp + carbamate + phosphoramidate aqueous solution after 16 h at 100°C**

pH measurements:

After 16 h: 10.6

NMR data:


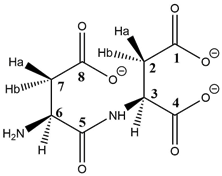


^1^H NMR (Bruker, 500.07 MHz, D_2_O, 25 °C, ppm)

β-Asp-Asp: δ 4.11 (dd, 7.6 Hz, NH), δ 2.96 (dd, 12.3 Hz, 8.2 Hz, H_3_), δ 2.55 (dd, 8.6 Hz, 4.1 Hz, H_6_), δ 1.54 (dd, 16.4 Hz, 4.1 Hz, H_2a_), δ 1.43 (dd, 14.8 Hz, 4.4 Hz, H_7a_), δ 1.35 (dd, 16.3 Hz, 8.5 Hz, H_2b_), δ 1.26 (dd, 14.8 Hz, 8.8 Hz, H_7b_)

Asp: minor, mostly hidden by β-Asp-Asp

H_2_O: δ 3.85 (s)

**
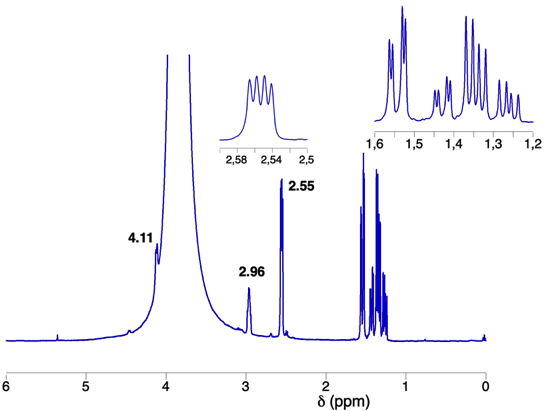
**

**Fig. S15** ^1^H NMR spectrum of Asp + carbamate + phosphoramidate aqueous solution after 16 h at 100 °C

^13^C NMR (Bruker, 125.74 MHz, H_2_O, 25 °C, ppm):

Carbamate: δ 165.41 (s)

Hydrogenocarbonate: δ 163.64 (s)

β-Asp-Asp: δ 178.6 (s, C1 and C8), δ 178.2 (s, C4), δ 165.1 (s, C5), δ 54.9 (s, C3), δ 54.9 (s, C6), δ 40.8 (s, C2), δ 39.9 (s, C7)

Asp: δ 180.9 (s, CO_2_^-^), δ 179.5 (s, CO_2_^-^), δ 52.9 (s, CH), overlapped δ 39.9 (s, CH_2_)

**
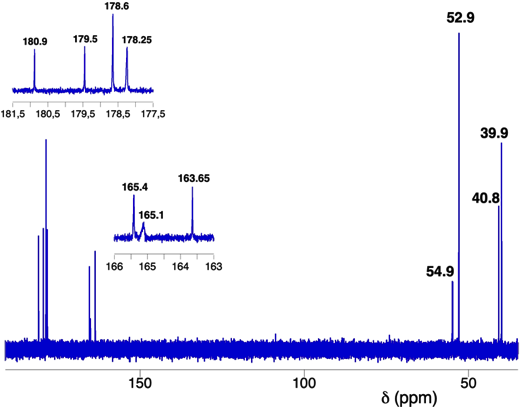
**

**Fig. S16** ^13^C NMR spectrum of Asp + carbamate + phosphoramidate aqueous solution after 16 h at 100 °C

**9) Asp + carbamoyl donors on silica at 25 °C**

**
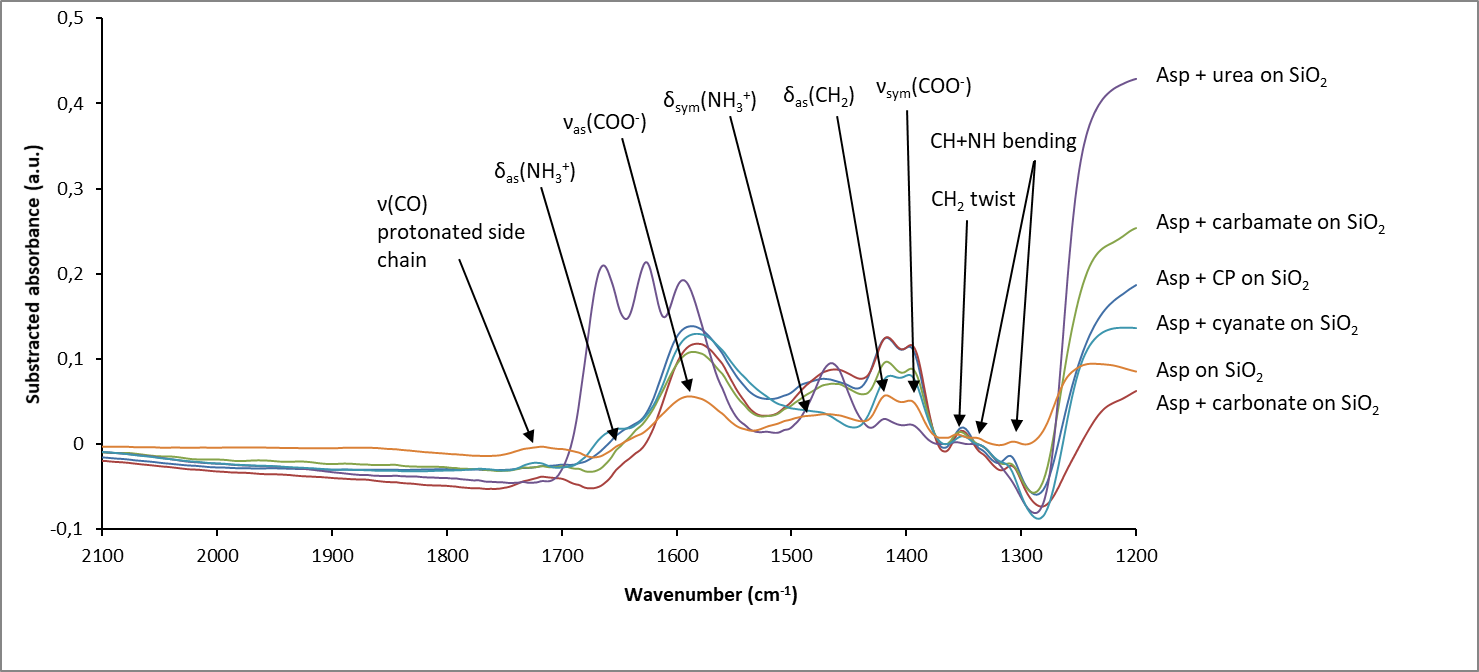
**

**Fig. S17** Stacked transmission-IR spectra of aspartic acid deposited with carbamoyl donors on amorphous at 25 °C

Raw silica IR spectrum was subtracted to only see the organic bands.

**10) Mg(Asp)_2_ on magnesium carbonate at 25 °C**

NMR data after desorption in D_2_O:

^1^H NMR with solvent suppression (Bruker, 500.07 MHz, D_2_O, 25 °C, ppm)

Aspartate: δ 3.70 (dd, 3.6 Hz, 8.31 Hz, 1H, H_α_); 2.69 (dd, 3.9 Hz, 8.3 Hz, 1H, H_β1_); 2.52 (dd, 8.5 Hz, 17.2 Hz, 1H, H_β2_)

**
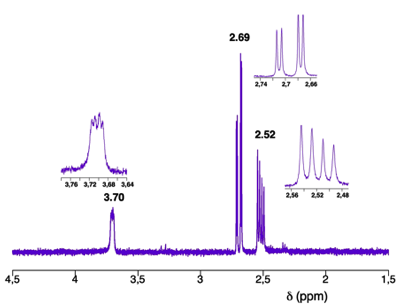
**

**Fig. S18** ^1^H NMR spectrum of Mg(Asp)_2_ on magnesium carbonate at 25 °C after desorption in D_2_O

XRD data:

pXRD (Bruker, Cu Kα_1_, 5-80°, step size: 0.0205°/datapoint, step time: 1 s/datapoint)

The diffraction peaks matching with hydromagnesite phase (4(MgCO_3_). (Mg(OH)_2_) • 4 H_2_O). No trace of crystalline organic matter.


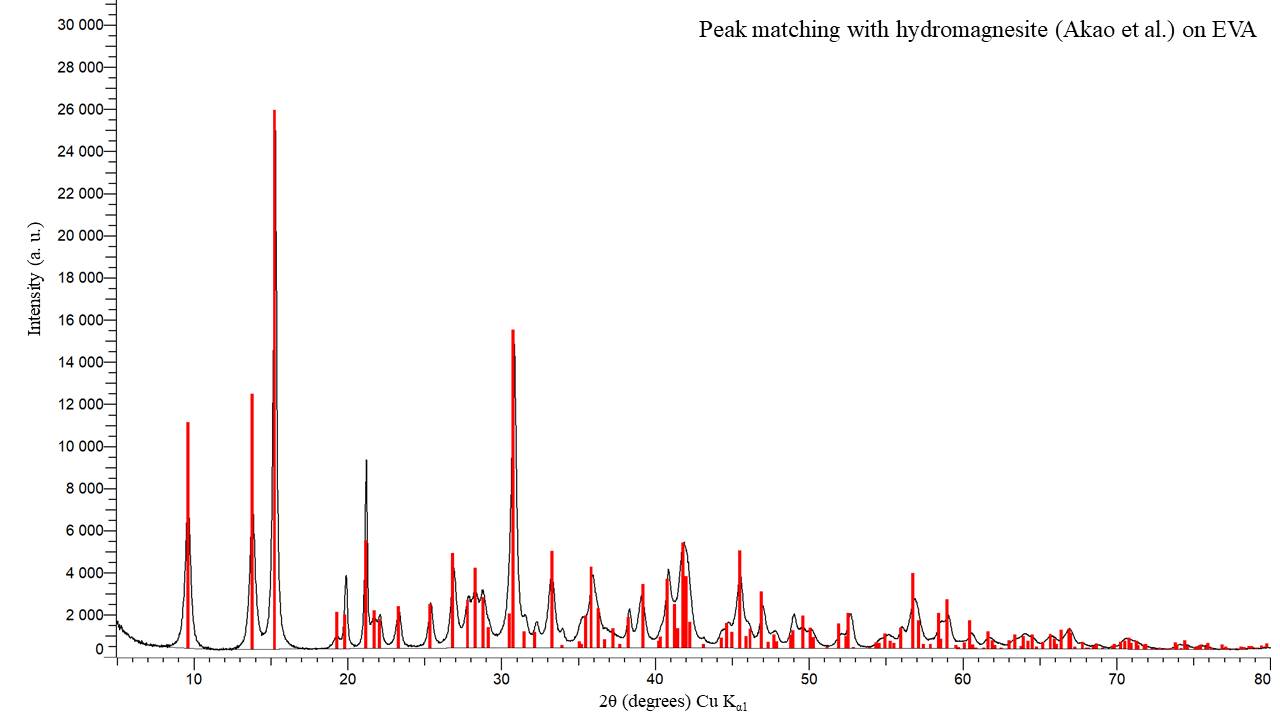


**Fig. S19** Powder diffractogram of magnesium carbonate after water impregnation and drying at 25 °C


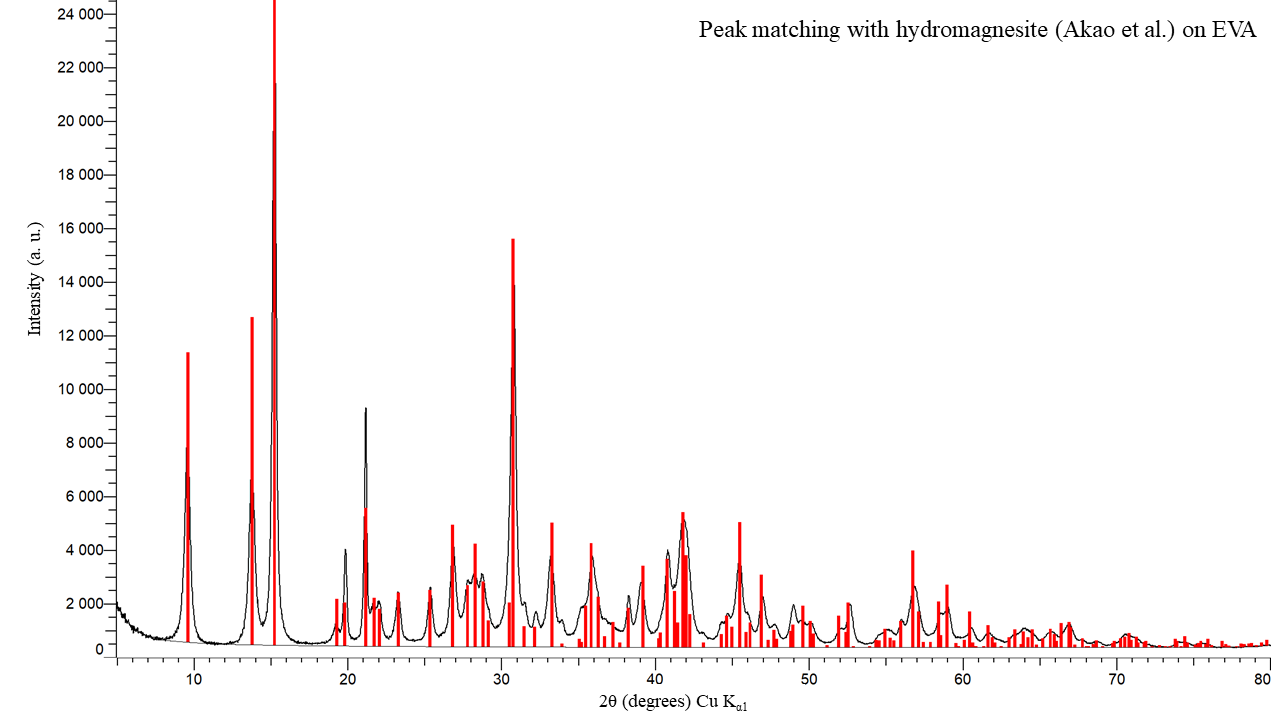


**Fig. S20** Powder diffractogram of Mg(Asp)_2_ on magnesium carbonate at 25 °C

**11) Asp-Asp on magnesium carbonate at 25 °C**

NMR data:

^1^H NMR (Bruker, 500.07 MHz, D_2_O, 25 °C, ppm)


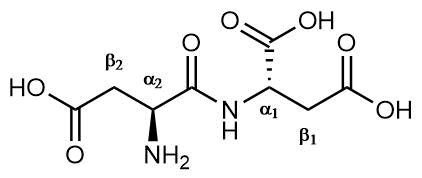


Asp-Asp dimer: δ 4.34 (dd, 4.2 Hz, 9.2 Hz, 1H, H_α1_); 3.69 (dd, 4.2 Hz, 9.4 Hz, 1H, H_α2_); 2.62 (m, 4.3 Hz, 15.6 Hz, 2H, H_β1_ and H_β2_); 2.49 (dd, 9.2 Hz, 15.2 Hz, 1H, H_β1_), 2.31 (dd, 9.2 Hz, 15.7 Hz, 1H, H_β2_)


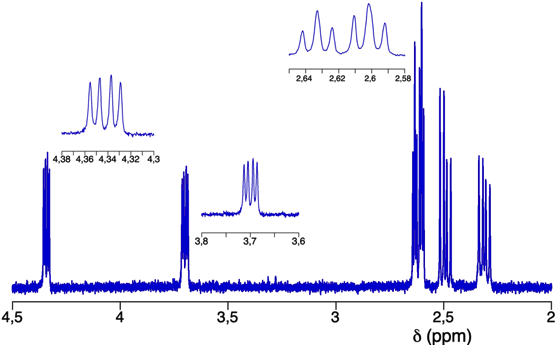


**Fig. S21** ^1^H NMR spectrum of Asp_2_ linear dimer on magnesium carbonate at 25 °C after desorption in D_2_O

XRD data:

pXRD (Bruker, Cu Kα_1_, 5-80°, step size: 0.0205°/datapoint, step time: 1 s/datapoint)

The diffraction peaks are matching with hydromagnesite phase (4(MgCO_3_). (Mg(OH)_2_) • 4 H_2_O). No trace of crystalline organic matter (cf. Figure S19).


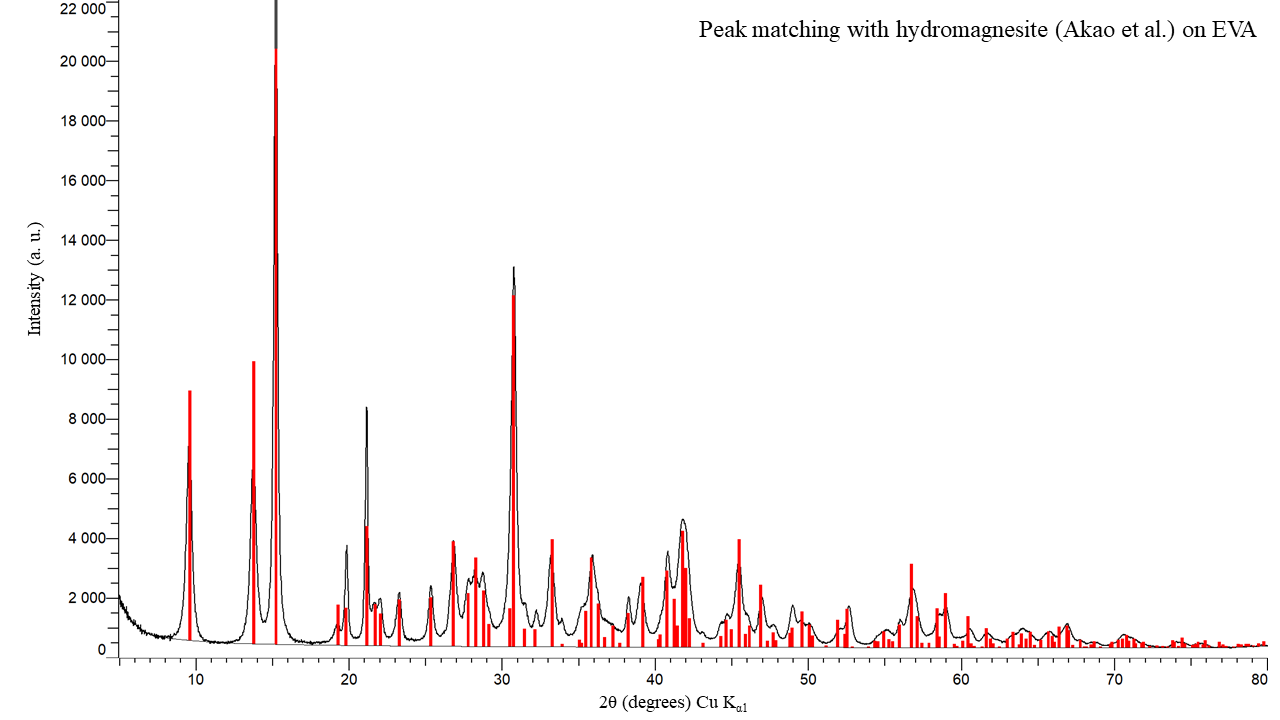


**Fig. S22** Powder diffractogram Asp_2_ linear dimer on magnesium carbonate at 25 °C

**12) NCA reference spectra**

NMR data after deposition on MgCO_3_ and desorption in D_2_O:

^1^H NMR with solvent suppression (Bruker, 500.07 MHz, D_2_O, 25 °C, ppm)

N-carbamoylaspartate: δ 4.11 (d(?), 7.6 Hz, H_α_), 2.55 (dd, 3.6 Hz, 15.9 Hz, 1H, H_β1_); 2.33 (dd, 9.7 Hz, 15.2 Hz, 1H, H_β2_).

NMR data of NCA in saturated magnesium carbonate solution:

^1^H NMR with solvent suppression (Bruker, 500.07 MHz, D_2_O, 25 °C, ppm)

N-carbamoylaspartate: δ 4.16 (dd, 3.7 Hz, 9.1 Hz, H_α_), 2.59 (dd, 4.0 Hz, 15.4 Hz, 1H, H_β1_); 2.39 (dd, 9.3 Hz, 15.4 Hz, 1H, H_β2_).


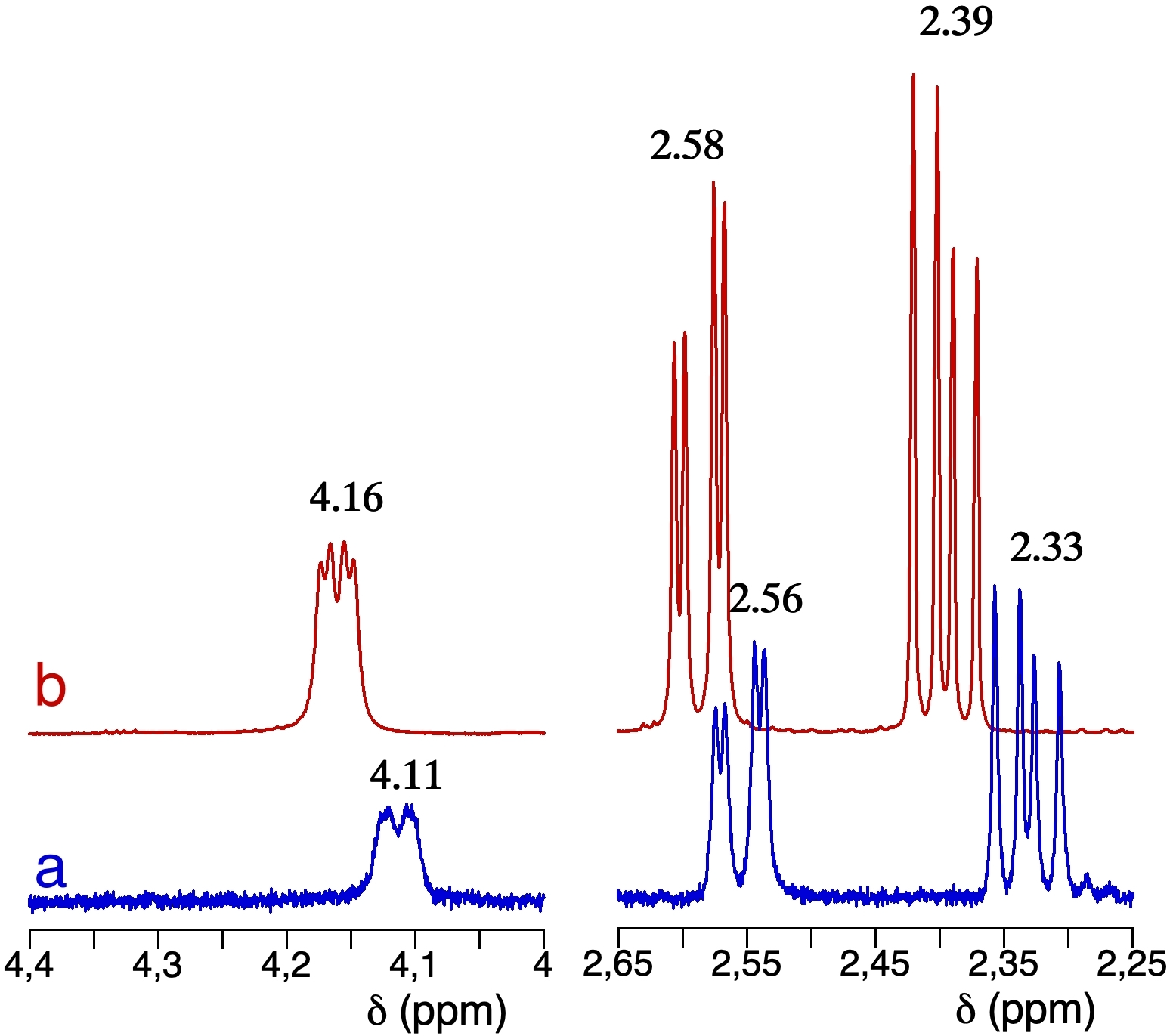


**Fig. S23** ^1^H NMR spectrum of a) NCA on magnesium carbonate at 25 °C after desorption in D_2_O, b) NCA in saturated magnesium carbonate solution at 25 °C in D_2_O.

XRD data of NCA/MgCO_3_:

pXRD (Bruker, Cuα_1_, 5-80°, step size: 0.0205°/datapoint, step time: 1 s/datapoint)

The diffraction peaks match with the hydromagnesite phase (4(MgCO_3_). (Mg(OH)_2_) • 4 H_2_O). No trace of crystalline organic matter.


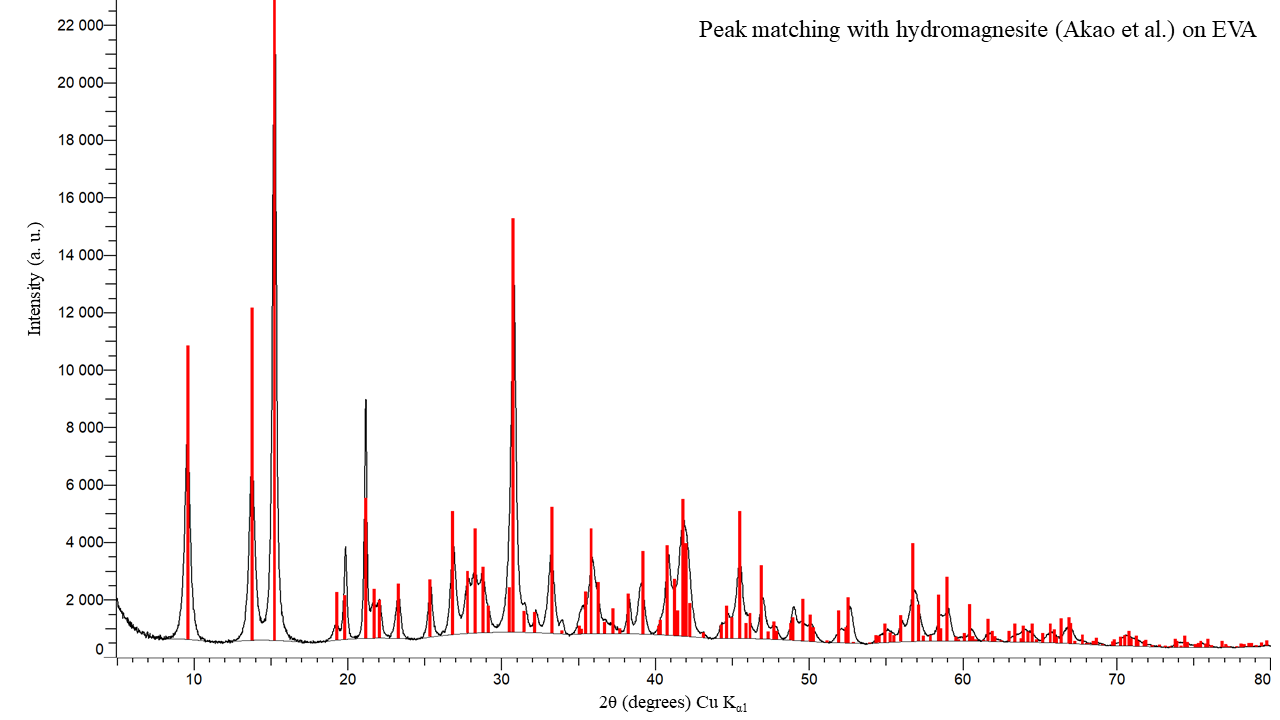


**Fig. S24** Powder diffractogram NCA on magnesium carbonate at 25 °C

**13) Mg(Asp)_2_ + sodium cyanate on magnesium carbonate at 25 °C**

pH measurements:

MgCO_3_ into 6 mL H_2_O: 10.44. Mg(Asp)_2_ + NaNCO into 6 mL H_2_O: 8.06. Mg(Asp)_2_ + NaNCO + 500 mg MgCO_3_ into 6 mL H_2_O: 9.08.

NMR data after desorption in D_2_O:

^1^H NMR with solvent suppression (Bruker, 500.07 MHz, D_2_O, 25 °C, ppm)

Aspartate: δ 3.65 (m, 1H, H_α_); 2.64 (dd, 3.8 Hz, 17.3 Hz, 1H, H_β1_); 2.46 (dd, 8.4 Hz, 16.5 Hz, 1H, H_β2_)

N-carbamoylaspartate: δ 4.12 (m, H_α_), 2.56 (dd, 3.6 Hz, 15.2 Hz, 1H, H_β1_); 2.33 (dd, 9.8 Hz, 15.2 Hz, 1H, H_β2_)


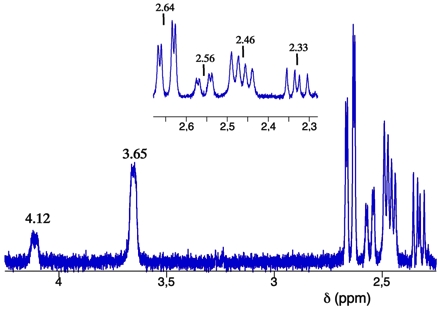


**Fig. S25** ^1^H NMR spectrum of Mg(Asp)_2_ + sodium cyanate on magnesium carbonate at 25 °C after desorption in D_2_O

XRD data:

pXRD (Bruker, Cuα_1_, 5-80°, step size: 0.0205°/datapoint, step time: 1 s/datapoint)

The diffraction peaks match with the hydromagnesite phase (4(MgCO_3_). (Mg(OH)_2_) • 4 H_2_O). No trace of crystalline organic matter.


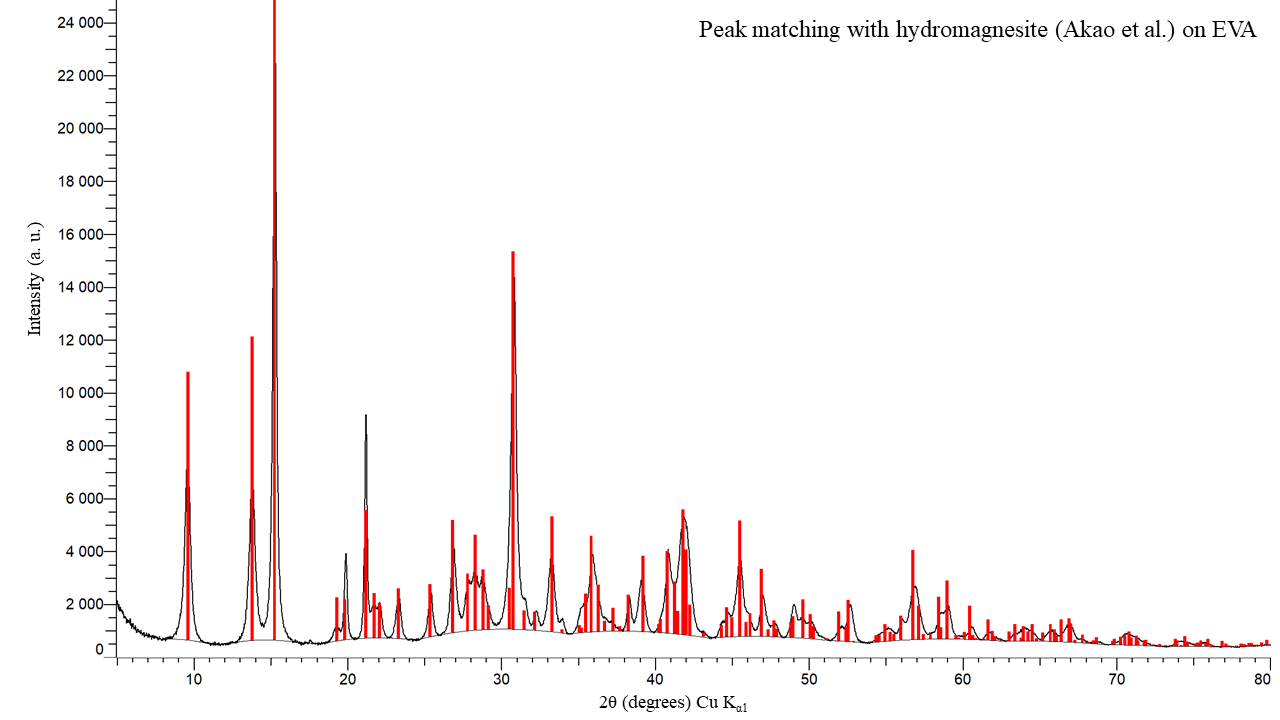


**Fig. S26** Powder diffractogram of Mg(Asp)_2_ + sodium cyanate on magnesium carbonate at 25 °C

**14) Mg(Asp)_2_ + sodium cyanate on magnesium carbonate at 150 °C**

NMR data after desorption in D_2_O:

^1^H NMR with solvent suppression (Bruker, 500.07 MHz, D_2_O, 25 °C, ppm)

Aspartate: δ 3.64 (m, 1H, H_α_); 2.64 (dd, 3.6 Hz, 17.0 Hz, 1H, H_β1_); 2.46 (dd, 8.3 Hz, 17.0 Hz, 1H, H_β2_)

N-carbamoylaspartate: δ 4.11(m, H_α_), 2.55 (dd, 3.6 Hz, 15.0 Hz, 1H, H_β1_); 2.33 (dd, 9.8 Hz, 15.2 Hz, 1H, H_β2_)


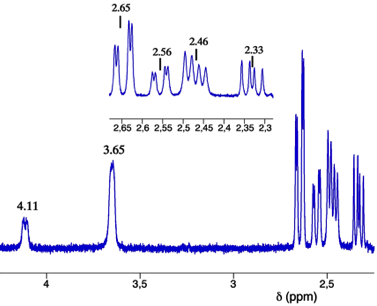


**Fig. S27** ^1^H NMR spectrum of Mg(Asp)_2_ + sodium cyanate on magnesium carbonate at 150 °C after desorption in D_2_O

**15) Mg(Asp)_2_ + sodium cyanate on magnesium carbonate at 200 °C**

NMR data after desorption in D_2_O:

^1^H NMR with solvent suppression (Bruker, 500.07 MHz, D_2_O, 25 °C, ppm)

Aspartate: δ 3.60 (m, 1H, H_α_); 2.62 (dd, 3.9 Hz, 16.9 Hz, 1H, H_β1_); 2.43 (dd, 8.4 Hz, 17.2 Hz, 1H, H_β2_)

N-carbamoylaspartate: δ 4.10 (m, H_α_), 2.54 (dd, 3.9 Hz, 15.4 Hz, 1H, H_β1_); 2.31 (dd, 9.8 Hz, 15.4 Hz, 1H, H_β2_)


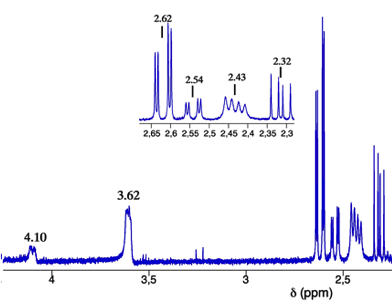


**Fig. S28** ^1^H NMR spectrum of Mg(Asp)_2_ + sodium cyanate on magnesium carbonate at 200 °C after desorption in D_2_O

**16) Mg(Asp)_2_ + sodium cyanate on magnesium carbonate at 230 °C**

NMR data after desorption in D_2_O:

^1^H NMR with solvent suppression (Bruker, 500.07 MHz, D_2_O, 25 °C, ppm)

Aspartate: δ 3.49 (dd, 4.2 Hz, 7.0 Hz, 1H, H_α_); 2.58 (dd overlapped with NCA, 4.2 Hz, 17.0 Hz, 1H, H_β1_); 2.31 (dd, 9.7 Hz, 15.4 Hz, 1H, H_β2_)

N-carbamoylaspartate: δ 4.10 (dd (?), H_α_), 2.54 (dd overlapped, 3.7 Hz, H_β1_); 2.16 (dd, 10.2 Hz, 15.7 Hz, 1H, H_β2_)

Degradation products: δ3.25 (s); 3.21 (s); 2.41-2.38; 1.78 (s)


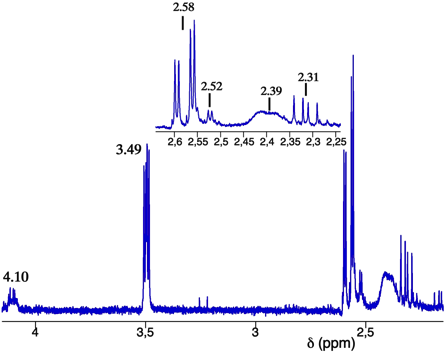


**Fig. S29** ^1^H NMR spectrum of Mg(Asp)_2_ + sodium cyanate on magnesium carbonate at 230 °C after desorption in D_2_O

**17) Mg(Asp)_2_ + biuret on magnesium carbonate at 25 °C**

NMR data after desorption in D_2_O:

^1^H NMR with solvent suppression (Bruker, 500.07 MHz, D_2_O, 25 °C, ppm)

Aspartate: δ 3.70 (m, 1H, H_α_); 2.69 (dd, 3.9 Hz, 17.3 Hz, 1H, H_β1_); 2.52 (dd, 8.3 Hz, 17.3 Hz, 1H, H_β2_)

**
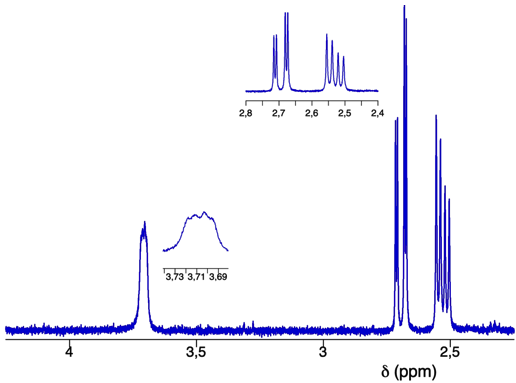
**

**Fig. S30** ^1^H NMR spectrum of Mg(Asp)_2_ + biuret on magnesium carbonate at 25 °C after desorption in D_2_O

**18) Mg(Asp)_2_ + biuret on magnesium carbonate at 140 °C**

NMR data after desorption in D_2_O:

^1^H NMR with solvent suppression (Bruker, 500.07 MHz, D_2_O, 25 °C, ppm)

Aspartate: δ 3.69 (m, 1H, H_α_); 2.69 (dd, 3.9 Hz, 17.3 Hz, 1H, H_β1_); 2.52 (dd, 8.0 Hz, 17.0 Hz, 1H, H_β2_)

**
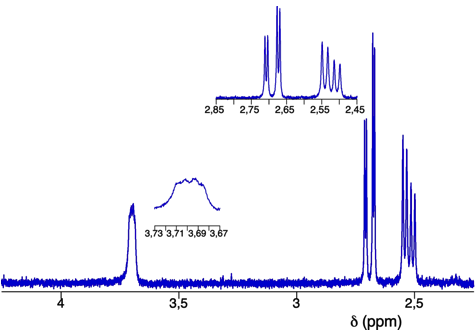
**

**Fig. S31** ^1^H NMR spectrum of Mg(Asp)_2_ + biuret on magnesium carbonate at 140 °C after desorption in D_2_O
